# Public Health Impact and Cost-Effectiveness of 2-Dose vs 1-Dose Human Papillomavirus Vaccination Regimen in Saudi Arabia

**DOI:** 10.36469/001c.160028

**Published:** 2026-04-23

**Authors:** Abdullah M. Assiri, Carole Mamane, Dzhumber Ugrekhelidze, Laurie Lévy-Bachelot, Haleema A.M. Alserehi, Ghadah Albassam, Ahmed S. Bassyouni, Tufail M. Malik, Vincent Daniels

**Affiliations:** 1 Deputyship of Population Health Ministry of Health, Riyadh, Saudi Arabia; 2 Outcomes Research, Eastern Europe Middle East and Africa, MSD, Puteaux, France; 3 Health Economic and Decision Sciences, MSD Switzerland, Lucerne, Switzerland; 4 MSD, France, Puteaux, France; 5 4Health Economics and Outcomes Research, Gulf Cooperation Council, MSD Scientific Office, Riyadh, Saudi Arabia; 6 Medical Affairs Gulf Cooperation Council, MSD Scientific Office, United Arab Emirates; 7 Health Economic and Decision Sciences Merck & Co., Inc., Rahway, New Jersey, USA

**Keywords:** human papillomavirus, cost-effectiveness, public health impact, Saudi Arabia, single-dose vaccine

## Abstract

**Background:**

The Saudi Food and Drug Authority recently registered a 9-valent human papillomavirus (HPV) vaccine, which provides broader protection than the existing 4-valent vaccine against genital warts and various cancers. A single-dose protocol vs a 2-dose protocol has been considered as an option for girls aged 9 to 14 and 15 to 20 years in the Kingdom of Saudi Arabia (KSA).

**Objective:**

To assess cost-effectiveness and health outcomes associated with 2-dose vs 1-dose 9-valent HPV (9vHPV) vaccination in the KSA.

**Methods:**

A dynamic transmission model was used to assess public health impact and incremental cost-effectiveness ratio (ICER) of a 2-dose compared with a 1-dose 9vHPV program. Costs (2023 SAR) and quality-adjusted life-years (QALY) were discounted at 3%. Vaccination coverage was estimated using the Ministry of Health school-based program. Scenario analyses considered higher cervical cancer incidence and higher 1-dose vaccine effectiveness.

**Results:**

A 2-dose vs a 1-dose 9vHPV vaccine prevents 13 700 additional cases of HPV-related diseases over a time horizon of 100 years. Furthermore, the model yields an ICER of SAR 30 400/QALY gained. Under scenarios of higher cervical cancer incidence and higher 1-dose vaccine effectiveness, ICERs are SAR 17 400/QALY and SAR 83 200/QALY, respectively. For the scenario with higher cervical cancer incidence (ie, 6.1 per 100 000 women), the 2-dose program achieves cervical cancer elimination within a median time of 54 years, whereas the 1-dose program takes more than 100 years. In sensitivity analyses, the ICER remains below 1×GDP per capita (SAR 129 000).

**Conclusions:**

A 2-dose program shows a positive impact in terms of public health and cost-effectiveness compared with a 1-dose program.

## INTRODUCTION

Human papillomavirus (HPV) causes infections in the anogenital and upper airway regions and several types of cancers such as cervical cancer, cervical Intraepithelial neoplasia, vulvar cancer, vulvar intraepithelial neoplasia, vaginal cancer, vaginal intraepithelial neoplasia, penile, anal, and some head and neck cancers, as well as genital warts and recurrent respiratory papillomatosis.^1^ In particular, HPV types 16, 18, 31, 33, 45, 52, and 58 are 7 high-risk HPVs that cause about 90% of cervical cancers.^2^

Cervical cancer is of particular concern, ranking as the fourth most prevalent cancer type among women worldwide, with an estimated 604 000 new cases reported in 2020 according to the World Health Organization (WHO).^3^ In the Kingdom of Saudi Arabia (KSA), 358 new cases of cervical cancer and 179 deaths due to its complications were reported in 2020 alone, giving it a rank of the eighth most common malignancy in the country.^4^ The economic burden of cervical cancer and other HPV-related diseases has been reported as direct costs per episode of care in the literature. For cervical cancer, these costs were as high as SAR 75 963.00, SAR 89 852.90, and SAR 96 145.90 for local, regional and distant stages, respectively.^5^ Another study assessed the indirect cost of premature deaths by estimating productivity losses from HPV-related and hepatitis B–related cancer mortality in the Middle East and North Africa. In this study, Saudi Arabia reported $484 899.00 per death as value of years of life lost in terms of productivity loss due to HPV-related and hepatitis B–related cancer mortality.^6^

The 4-valent HPV vaccination prevents infection with types 6, 11, 16, and 18 and thereby can decrease the risk of cervical, vulvar, vaginal, and anal cancer caused by HPV types 16 and 18 and genital warts caused by HPV types 6 and 11.^7,8^

The 4-valent HPV vaccine was registered by the Saudi Food and Drug Authority (SFDA) in 2010 and is now provided free of charge as part of the school-based and community-based vaccination programs which were introduced end of March 2022. Moreover, insurance companies in the private sector are covering HPV vaccine as per the age groups approved in the Ministry of Health Program (2 doses for girls aged 9-14 years and 3 doses for females aged >15 years).^9^ More recently, the SFDA registered the 9-valent vaccine in January 2024.^10^

The school-based program was implemented with the first wave of vaccination targeting seventh-grade girls (ages 12-13), with an estimated cohort size of 250 000. The school-based program was announced to include 2 doses as per the product-approved label with a between-dose duration of 6 to 12 months for seventh-grade girls (12-13 years old). To date, the reported vaccination coverage rate (VCR) for the school-based program is 52%, with a cumulative VCR of 70% for the totality of the first wave.^11^ The 9-valent HPV vaccine offers broader protection than the 4-valent vaccine, where it extends its protection to include 5 additional high-risk HPV types—31, 33, 45, 52, and 58—providing increased protection against a wider array of HPV types that contribute to genital warts, 90% of cervical cancers and other cancers such as anal, oropharyngeal cancer, penile cancer, vaginal cancer, and vulvar cancer.^12,13^ It is believed that sustained comparable coverage rates for additional waves will be required to arrive at a well-established program that can provide the intended protection.

There is no nationally adapted screening or surveillance program to assess the prevalence of HPV-related diseases in KSA. To date, published data results are based on individual studies, most of which have concluded that the prevalence of HPV infection in their work and in the KSA overall, is likely underreported.^14^ For example, AlObaid et al found the overall prevalence of HPV to be 9.8% in women aged 15 years and older who were attended routine gynecological examinations.^15^ According to the HPV Information Center, data is not yet available on the HPV burden in the general population of KSA.^16^ Despite ongoing efforts, data on the prevalence of HPV are scarce in KSA and the true prevalence of HPV cannot be estimated until a national study is conducted covering all 13 provinces.^15^

In addition to the lack of surveillance program, the efficacy of the single dose has recently been tabled as potential option vs 2 doses in 9- to 14-year-old and 15- to 20-year-old girls.

Data from immunogenicity trials, post hoc analyses of efficacy trials, and post-licensure observational studies among females showed that the single dose regimen might be a comparable option to the 2 and 3 doses regimen.^17^ In 2022, the WHO updated the Strategic Advisory Group of Experts (SAGE) guidelines, recommending either a one- or two-dose regimen for HPV vaccination. However, the WHO has highlighted that further evidence should be generated on the long-term immunogenicity, efficacy, effectiveness, and duration of protection of single-dose HPV schedules in girls aged 9 to 14 years, boys, older women and men, and children less than 9 years of age. Findings from modeling studies indicate that, compared with a 2-dose regimen, a 1-dose regimen where 1 dose is not as effective as 2 doses will lead to occurrence of additional vaccine-preventable cases of HPV-related disease.^18–23^

With KSA’s focus on value-based healthcare, there is a pressing need for epidemiological and cost-effectiveness modeling to inform public health strategies regarding HPV vaccination schedules.^24^ More particularly, based on the available clinical evidence regarding the noninferiority of a single-dose HPV vaccine compared with a 2-dose schedule,^25–27^ there is a critical need to assess the long-term implications such as long-term duration of protection, population-level effects on HPV-related disease incidence and cost-effectiveness in KSA. To address these existing knowledge gaps, the current study adapted a previously published dynamic transmission model of HPV to evaluate the cost-effectiveness and health outcomes associated with a 2-dose vs 1-dose regimen of HPV vaccination in KSA. The modeling evidence, particularly the time needed to reduce cervical cancer rates, allows to assess whether a public health approach with the 2-dose program ensures an optimal protection of the population vs a 1-dose program, which was the status quo in terms of dose supply at the time of the study.

## METHODS

### Model Overview

**Figure 1.**
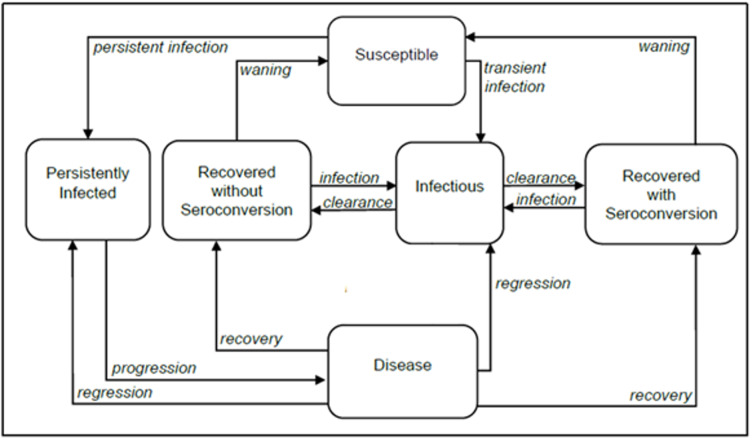
Simplified Schematic Diagram of the Compartmental Model of Human Papillomavirus Infection and Disease

We adapted a previously published compartmental deterministic dynamic transmission model of HPV infection and disease progression^18,28,29^ to evaluate the impact of a 2-dose vs a 1-dose regimen of 9vHPV vaccination on health and cost-effectiveness outcomes in KSA (**Figure 1**).

The model included all major HPV disease-related endpoints (ie, cervical, vaginal, and vulvar cancers and precancers, anal, penile, oropharyngeal cancers and genital warts), and key disease transitions, such as HPV acquisition, transmission, recovery, reinfection, natural immunity, progression to pre-cancerous lesions, development of cancer, disease detection, treatment, regression, and vaccination. A detailed description of the original model including the model structure, model calibration and validation was previously published.^29^

Key model outcomes were the number of HPV-related disease cases avoided, the incremental cost ratio (ICER) per quality-adjusted life-year (QALY) gained, and time to cervical cancer elimination. Model outcomes were assessed over a 100-year time horizon using ordinary differential equations, which were solved using the NDSolve function in Mathematica® 12 (Wolfram Research). In the base-case model, health utilities and costs were discounted at an annual rate of 3%.

### Model Inputs and Calibration

Key inputs and assumptions, including major cost inputs, vaccine efficacy parameters, duration of protection, and waning assumptions are described in **Table 1**. Details are provided in the **Supplementary Material.**

**Table 1.** Key Model Parameters

**Model Parameter**	**Inputs and Assumptions**
Vaccination strategy	Comparison of 2 vaccination strategies for girls-only vaccination (age 12-13): Status quo: 1-dose program Standard of care: 2-dose program
Vaccination coverage	VCR, first dose 52.0% in 2022 (school program data) 63.0% in 2023 (school program data) Peak of 76.0% by 2028: assumption based on mid-level coverage scenario defined by Bruni et al VCR, second dose: 67.0%, as estimated by Bruni et al regarding the mid-level coverage scenario
Vaccine effectiveness:	Degree of protection and duration of protection; 1-dose: Median relative vaccine effectiveness Against HPV 6/11/16/18: 98.4% Against HPV 31/33/45/52/58: 94.4% Median duration of protection: Against HPV 6/11/16/18: 9.1 years Against HPV 31/33/45/52/58: 8.3 years Probabilistic distributions for 1-dose vaccine degree and duration of protection: Base case analysis: data derived from Bayesian analysis of 18-month interim results from the KEN-SHE trial Scenario analysis: data from the India IARC study data Assumptions on duration of protection: 1-dose: assumed to wane over time following a gamma distribution for mean time to loss of protection 2-dose: assumed to provide lifelong protection
Cost inputs	Currency: SAR 2023 Discount rate: 3.0% Cost categories: Vaccine cost per dose: SAR 605.00 Administration cost: SAR 15.38 Cost of screening and diagnostic tests: Pap smear; SAR 511.00, colposcopy; SAR 635.00, biopsy: SAR 1062.00 Cost per episode of care for HPV-related diseases for genital warts and HPV related-cancers: Cervical, vaginal, vulvar, anal, penile and head and neck (costs provided per disease stage in **Supplementary Table S8**). Perspective: National single payer over 100 years
Utility inputs	Age-specific health utility values in the healthy population: based on international EQ-5D scores Health utility values for HPV-related disease: Based on US values (no Saudi values available)

Abbreviations: EQ-5D, EuroQol- 5 Dimension; HPV, human papillomavirus; SAR, Saudi Riyal; VCR, vaccination coverage rate.

### Demographics and Sexual Behavior

The population of KSA was set to 35 844 913, including 15 142 149 females and 20 702 764 males, as indicated in publicly available data.^30^ Rates of all-cause mortality by age and sex for KSA were obtained from data published by the WHO (**Supplementary Table S1**).^31^ Estimates of sexual behaviors were derived from surveys conducted in KSA (**Supplementary Tables S2 and S3**).^32–34^

### HPV Natural History

The model accounted for 9vHPV strain–attributable diseases. Based on clinical data from Serrano et al^12^ on genotype attribution for HPV 6, 11, 16, 18, 31, 33, 45, 52, and 58 in female anogenital lesions, the model assumed that 91.2% of HPV-positive cervical cancers involved infection with HPV-16, 18, 31, 33, 45, 51, 52, and 58 (**Supplementary Table S4**). The role of HPV coinfection was not assessed in the model. Mortality rates for HPV-related cancers varied with cancer stage, as presented in **Supplementary Table S5**.

### Vaccine Effectiveness

Vaccine effectiveness was represented by two parameters: the degree of protection and the duration of protection. A 2-dose regimen administered for ages less than 15 years was assumed to provide lifelong protection and have efficacy that varied by disease, endpoints, and sex, as reflected in clinical trial findings previously described.^18^ For clinical data on the effectiveness of a 1-dose regimen, we used KEN SHE trial^35^ data modeled a joint probability distribution of the degree of protection and duration of protection of 1-dose vaccination, which was assumed to wane over time following a gamma distribution for mean time to loss of protection. For HPV 6/11/16/18, the median relative effectiveness of a 1-dose compared with a 2-dose regimen was estimated to be 98.4%, and the median duration of protection, 9.1 years. For HPV 31/33/45/52/58, the median relative efficacy of a 1-dose compared with a 2-dose regimen was estimated to be 94.4%, and the median duration of protection 8.3 years. Details of the derivation of these values and the corresponding uncertainty distributions are described by Daniels et al.^18^

### Screening Patterns and Vaccination Coverage Rates

Note that KSA does not have a routine cervical screening program. However, based on survey data from KSA,4 we assumed that 15.0% of women aged 25 to 65 years had ever been screened, among whom the annual probability of being screening was assumed to be 2.4%. We further assumed that 72.5% of women received follow-up care following a positive cervical cancer screen, based on a recent study on cervical screening patterns (**Supplementary Table S6**).^36^ Women who performed hysterectomy were excluded from the model regarding cervical cancer screening and progression to cervical disease. Hysterectomy rates for each age group were based on published data (**Supplementary Table S7**).^37^

Annual (ie, not cumulative) VCRs for HPV vaccination were set to 52.0% for 2022 and 63.0% for 2023, as observed in school program data for 12-to-13-year-olds in KSA (**Supplementary Table S6**).^11^ VCRs were assumed to reach a peak of 76.0% by 2028, as per mid-level coverage definition according to Bruni et al.^38^ The VCR for completion of the second dose was assumed to be 67.0%, as estimated by Bruni et al regarding the mid-level coverage scenario (76.0% receives ≥1 dose, and 67.0% of those complete the 2-dose series). These estimations were based on coverage rates of 107 WHO member states that had introduced a National HPV Immunization Program from 2010 to 2019.

### Health Economics Parameters

Cost parameters, including costs for the treatment of stage-specific HPV-related cancer, HPV vaccination, and cervical cancer screening, were derived from the opinions of health experts in KSA (**Supplementary Table S8**). The modeled costs included vaccination costs for 9-valent HPV (9vHPV; SAR 605.00), estimated using public data,^39^ and costs associated with cervical cancer screening (ie, PAP smear and office visit, SAR 511.00; colposcopy, SAR 635.00; and biopsy, SAR 1062.50). Health utility values refer to the QALY, which has a value between 0 and 1. Health utility values for HPV-related disease were based on a previous model from the United States.^40^ Age-specific health utility values in the healthy population were based on previously international published EQ-5D scores (**Supplementary Table S9**).^41^

The cost-analysis used modeled values for distribution of the burden of HPV-related diseases in KSA, including HPV-related cancer incidence (**Supplementary Table S10**) and mortality (**Supplementary Table S11**).

### Calibration

Using baseline parameter inputs, HPV-related disease models were separately calibrated by minimizing the residual function of the model outcomes and certain targeted real-world outcome data to determine best fitting model parameter values, as described in the **Supplementary Material**.

### Model Outputs

Model outputs included epidemiological outcomes such as total and incremental cases of HPV-related cancers and time to cervical cancer elimination comparing a 2-dose program with a 1-dose 9vHPV vaccination program (ie, health outcomes). The model also estimated mean discounted costs, mean discounted QALYs and thus, predicted a mean ICER. Cost-effectiveness acceptability curves were constructed to estimate the probability that 1-dose or 2-dose option was cost-effective according to the willingness to pay (WTP). Cost and QALY for the 1-dose and 2-dose HPV vaccination programs were calculated according to the methodology described in Daniels et al.40 Cost-effectiveness assessments were based on the WTP of 1 gross domestic product (GDP) per capita in KSA, equivalent to $34 454.212 in 2022 (129 223.11 SAR).^42^

All results were obtained from statistical distributions resulting from probabilistic sensitivity analysis (PSA) modeling single-dose duration and degree of protection based on empirical distributions of these vaccine parameters. We used 1000 samples from an empirical distribution derived from the KEN SHE trial data for the base case analysis and from the India International Agency for Research for Cancer (IARC) study data as scenario analysis.

### Additional Scenario Analysis

The calibrated model was utilized to forecast the incidence of cervical cancer and genital warts with 1-dose and 2-dose of 9vHPV vaccination programs spanning 100 years. These projections were drawn for a base case set of parameter values as described above in model inputs. However, additional analyses were carried out to account for (1) the uncertainty in the observed HPV prevalence and in the reported cervical cancer incidence and (2) the uncertainty in the vaccine effectiveness of the 1-dose program.

### Increase of Cervical Cancer Incidence

Surveillance data indicate a cervical cancer incidence in KSA equivalent to a crude rate of 2.44 per 100 000 women and an age-adjusted rate of 2.81 per 100 000 women. However, this incidence markedly increases with age: above the age of 55, the cervical cancer incidence in KSA becomes higher than in the United Kingdom for the same age categories.16,30 According to expert opinions, cervical cancer in KSA is underdiagnosed and underreported.^43^ Furthermore, findings from a published report indicate that the number of new cervical cancer cases in KSA increased by 453.6% from 1990 to 2019 (incidence multiplied by 4.5 in 30 years).^44^ Thus, to address the uncertainty regarding cervical cancer incidence in KSA, we conducted a scenario analysis in which we assumed higher burden estimates than reported values: 2-fold higher for the prevalence of genital HPV, 2.5-fold higher for the incidence of cervical cancers (ie, crude incidence per 100 000 women of 6.1), and 1.5-fold higher for mortality associated with HPV-related cancers. The age distribution of HPV-related cancer incidence was assumed to be unchanged. The scenario model was used to estimate the distribution of health outcomes and cost-effectiveness outcomes, comparing a 2-dose program to a 1-dose vaccination program. Time to elimination for the 2-dose vs 1-dose program was estimated according to the WHO elimination threshold of 4.0 per 100 000 for cervical cancer incidence.^45^

### Increase of 1-Dose Vaccine Effectiveness

An analysis was performed to assess the uncertainty around the 1-dose 9vHPV vaccine effectiveness. We considered a higher effectiveness of the 1-dose vaccination with an alternative distribution of the degree of protection and duration of protection of 1-dose vaccination based on 10-year follow-up data from the India IARC study.

### Sensitivity Analysis

One-way deterministic sensitivity analyses were conducted to evaluate the robustness of the cost-effectiveness model under various scenarios for a range of parameters:

- Discounting cost and QALYs by 0% or 5% (base case, 3%)
- **Uncertainty in 1-dose effectiveness parameters:** The analysis incorporates uncertainty in the effectiveness of the 1-dose program, drawing from the analysis of KENSHE and IARC India study data. This includes exploring variations in the mean duration of protection, with the base case reflecting a mean duration of protection of around 10 years, while the expanded analysis considers a mean duration of protection of about 20 years.
- **Varying all disease state utilities simultaneously by ± 20%:** The sensitivity analysis examines the impact of simultaneously varying disease state utilities by ± 20%, from a maximum utility of 1.0 to a minimum of 0, enabling an evaluation of the model’s response to changes in health utility values.
- **Varying dose price by ± 20% (base case price, SAR 620.83):** This sensitivity analysis explores the model’s response to the price of the vaccine dose, encompassing both an increase and decrease of ± 20% from the base case price of SAR 620.83 per dose.
- **Varying coverage target assumptions:** The analysis considers variations in coverage target assumptions, ranging from 63% (41% completion) to 90% (89% completion), with the base case reflecting a coverage target of 76% with 79% completion. This assessment evaluates the model’s sensitivity to changes in vaccination coverage levels and completion rates.
- **Varying all treatment costs simultaneously by ± 20%:** By simultaneously varying all treatment costs by ± 20%, the model’s response to variation in the costs associated with treatments for the target disease is examined, providing insights into the model’s sensitivity to changes in treatment costs.
- **Increasing target pre-vaccine cervical cancer incidence by a factor of 2.5:** This sensitivity analysis explores the impact on model outcomes by considering the target pre-vaccine cervical cancer incidence level to be 2.5 times that used in base case model calibration.

## RESULTS

### Health Outcomes

**Figure 2.**
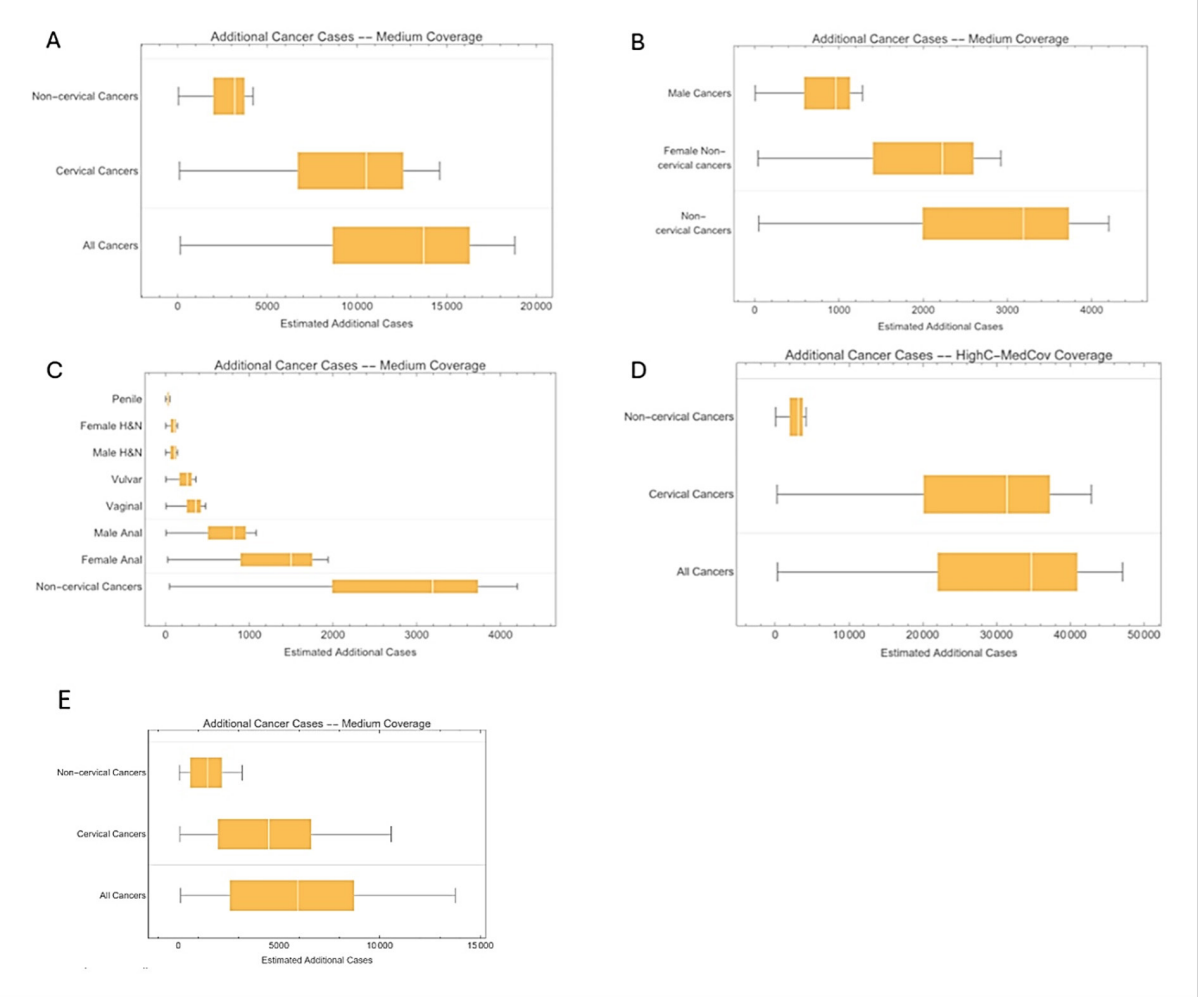
Estimated Number of HPV-Related Cancers Averted by a 2-Dose vs a 1-Dose Regimen Abbreviations: H&N, head and neck; HPV, human papillomavirus. All panels show the estimated number of additional cases averted by a 2-dose compared with 1-dose HPV vaccination. Assuming the base-case scenario, **Panel A** presents estimates for all cancer cases, including cervical and noncervical cancers; **Panel B** presents estimates for noncervical cancer cases overall and by gender; and **Panel C** presents estimates for subtypes of noncervical cancer. **Panel D** and **Panel E** report outcomes for the scenario analysis with higher cervical cancer incidence and higher 1-dose vaccine effectiveness, respectively. The boxes in the box plots represent the interquartile range (25%-75%), while the white vertical lines indicate the median, and the whiskers portray the minimum and maximum value.

**Base case analysis: Figure 2A-2C** illustrates the additional disease burden reduction achieved with a 2-dose 9vHPV vaccination program compared with a 1-dose program throughout the entire vaccination time horizon. The 1-dose program was less effective in preventing cases of cervical and noncervical cancers. With greater effectiveness and longer protection duration, the 2-dose program was projected to prevent an additional approximately 13 700 cases (95% CI 8600-16 400), leading to a more significant reduction in HPV-related disease burden, particularly cervical cancers. Among noncervical cancers, female cancers represented a substantial portion of those prevented (**Figure 2C**). Overall, the 2-dose 9vHPV program was expected to prevent more noncervical cancer cases on average than the 1-dose program, indicating its superior impact on noncervical cancer prevention.

In terms of cervical cancer elimination timelines, the first 40 years of vaccination showed similar outcomes for both programs. However, disparities between the 1-dose and 2-dose 9vHPV programs became increasingly evident after 40 years (**Figure 2A**). As time progressed, the median incidence of cervical cancer with the 1-dose program significantly exceeded that of the 2-dose program (~1.7 per 100 000 vs 0.4 per 100 000 after 100 years). However, uncertainty surrounding the 1-dose program increased over time, as illustrated by the expanding orange area in **Figure 2**, while the blue area for the 2-dose program remained narrower.

The cervical cancer incidence data focused on high-risk HPV types (HPV 16, 18, 31, 33, 45, 52, and 58). The initial incidence of approximately 2.4 per 100 000, though below the WHO’s elimination threshold of 4 per 100 000, was notably higher than the age-adjusted incidence for the overall population. This discrepancy arose from the demographic model, which underestimated middle-age population sizes while overestimating those in older age groups. The ongoing demographic shift in KSA suggests that the current peak in population distribution was temporary and will decline, causing initial overestimations of cancer incidence compared with reported figures in the first 10 to 20 years. Nonetheless, there was evidence of a rising trend in cervical cancer incidence over time, likely linked to this demographic shift.

**Scenario analysis: Figure 2D and 2E** correspond to **Figure 2A** but reflect different assumptions regarding higher cervical cancer incidence and greater 1-dose vaccine effectiveness. In both scenarios, the majority of additional cancer cases prevented by the 2-dose 9vHPV program were cervical cancers. However, in the higher incidence scenario (**Figure 2D**), the distinction between the two programs became more pronounced. The assumptions of increased 1-dose vaccine effectiveness in **Figure 2E** diminished the gap between the two programs. The estimated additional noncervical cancer cases in these alternative scenarios remained consistent with the base case as they maintained identical baseline incidences.

**Figure 3.**
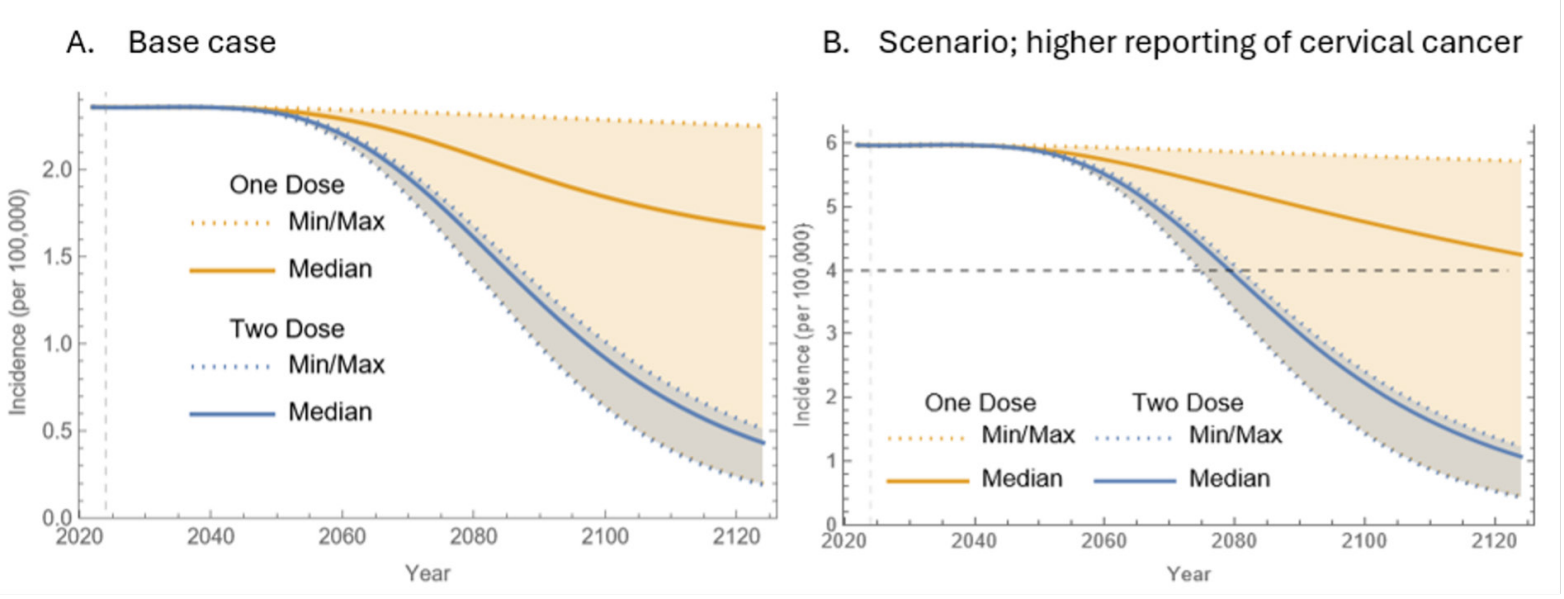
Projected Cervical Cancer Rates per 100 000 in KSA Over a 100-Year Period Abbreviation: KSA, Kingdom of Saudi Arabia. **Panel A** represents the base case analysis, and **Panel B** the scenario with higher reporting of cervical cancer incidence. In Panel B, the dashed line at the incidence of 4 per 100 000 women-years indicates the elimination threshold. This scenario analysis assumed a crude incidence of cervical cancer of 6 per 100 000 woman-years.

**Figure 3B** demonstrates cervical cancer incidence trends under assumptions of higher reporting. Under these conditions, the 2-dose program was projected to reach the elimination threshold of 4 cases per 100 000 between 2075 and 2080, with a median estimate in 2079. In comparison, the 1-dose program could potentially meet this threshold around 2075, given it performs as effectively as the 2-dose program, but would be unlikely to achieve elimination beyond 2125.

### Cost-Effectiveness Outcomes

**Figure 4.**
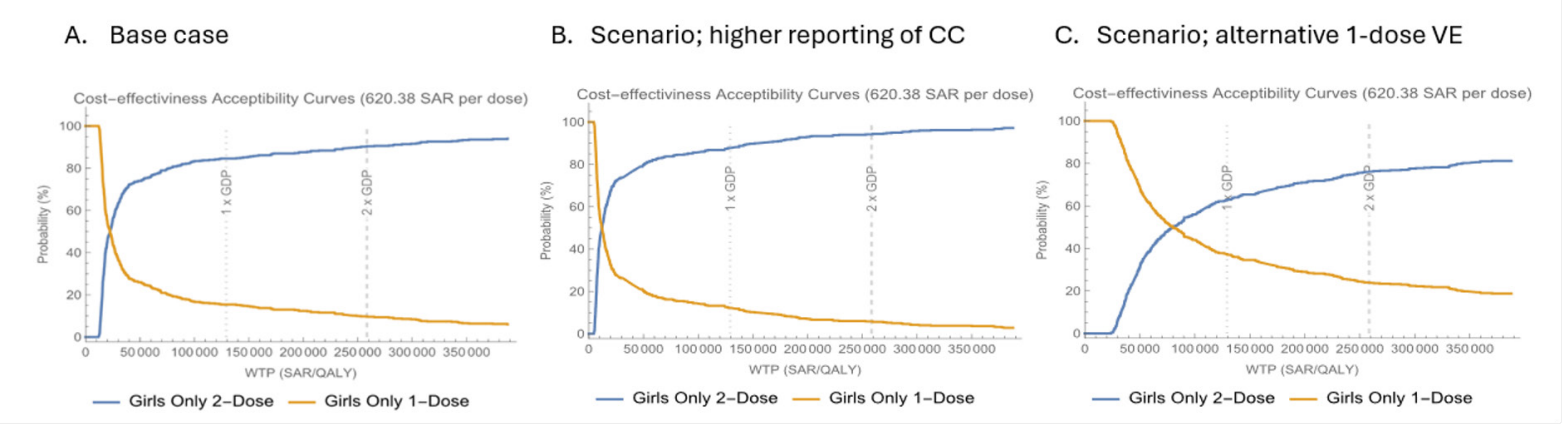
Cost-Effectiveness Acceptability Curve Comparing a 2-Dose vs a 1-Dose Regimen of HPV Vaccination Abbreviations: CC, cervical cancer; GDP, gross domestic product; HPV, human papillomavirus; QALY, quality-adjusted life-year; VE, vaccine effectiveness; WTP, willingness to pay. Cost-effectiveness acceptability curves are shown in orange for 1-dose strategy and in blue for 2-dose strategy under the base case (**A**) and the assumptions of “higher reporting” (**B**) and “alternative 1-dose vaccine effectiveness” (**C**). The vertical dashed lines represent the WTP thresholds for 1× and 2× GDP per QALY, respectively. The WTP threshold was set to 1 GDP per capita for KSA, which was 129 223.11 SAR (equivalent to $34 454.21 in 2022).

**Base case:** The 2-dose 9vHPV vaccination strategy for Saudi females was found to be cost-effective against the 1-dose program, with a predicted mean ICER of SAR 30 400/QALY (**Table 2**), considering a cost-effectiveness threshold of approximately SAR 129 000 per capita (1 GDP). **Figure 4A** displays the acceptability curves for the base case cost-effectiveness of both programs at a per-dose cost of SAR 620.38. The curves show that the 1-dose program is more likely to be cost-effective for WTP values below approximately SAR 22 000/QALY, while the 2-dose program is favored for values above this threshold. At the WTP threshold of 1 GDP, the 2-dose program has an 84% probability of being cost-effective.

**Table 2.** Mean Incremental Cost-Effectiveness of a 2-Dose vs a 1-Dose Regimen of 9vHPV Vaccination in KSA

**Strategy**	**Mean Costs (SAR)^a^**	**Mean QALYs**	**Δ Cost**	**Δ QALY**	**Mean ICER (SAR/QALY)**
Base-case scenario					
Girls only, 1-dose	421.41	28.0661			–
Girls only, 2-dose	451.88	28.0671	30.47	0.001002	30 400
Scenario analysis^b^					
Girls only, 1-dose	492.15	28.0563			
Girls only, 2-dose	517.42	28.0577	25.27	0.001450	17 400
Sensitivity analysis^c^					
Girls only, 1-dose	402.54	28.067065	–	–	–
Girls only, 2-⁠dose	444.45	28.067569	41.91	0.000504	83 200

Abbreviations: ICER, incremental cost-effectiveness ratio; SAR, Saudi Riyal; QALY, quality-adjusted life-year.

^a^All costs given values refer to 2023 SAR. Costs reflect total per person costs, including those associated with vaccination, HPV screening, and treatment of HPV-related disease.

^b^The scenario analysis assumed that the actual burden of cervical cancer was 2.5× higher than that reported in surveillance data.

^c^The scenario analysis assumed an alternative 1-dose with increased durability.

**Scenario analysis:** In the two alternative assumptions of higher cervical cancer incidence and higher 1-dose 9vHPV vaccine effectiveness, the 2-dose 9vHPV vaccination strategy remained cost-effective relative to the 1-dose strategy, with predicted mean ICERs of SAR 17 400/QALY and SAR 83 200/QALY, respectively **(Table 2). Figure 4B and 4C** illustrate the cost-effectiveness acceptability curves under these scenarios, demonstrating that the 1-dose program is more likely to be cost-effective at lower WTP thresholds (~SAR 12 000-13 000/QALY), while the 2-dose program is favored at higher thresholds. At the WTP of 1 GDP, the probability of the 2-dose program being cost-effective was estimated at 88% and 60% under the assumptions of higher cervical cancer incidence and higher 1-dose effectiveness, respectively.

### Cost-Effectiveness Sensitivity Analysis

The deterministic sensitivity analysis provided a detailed exploration of variations and uncertainties within the cost-effectiveness model, highlighting the robustness of its findings. A tornado diagram (**Supplementary Figure S12**) illustrates the most influential parameters. Throughout all variations, the ICER remained consistently below the 1 GDP threshold, indicating cost-effectiveness across sensitivity analyses.

The analyses showed that the mean ICER is most sensitive to variations in the discount rate, with significant differences observed at rates of 0%, 3%, and 5%. Following discount rate changes, the effectiveness of the 1-dose vaccine emerged as the next most sensitive factor. The model displayed varying ICER outcomes depending on whether effectiveness was based on KEN-SHE data alone or included both KEN-SHE and IARC India studies, resulting in mean protection durations of about 10 years and 20 years, respectively. Additionally, variations in utility values significantly influenced the ICER, underscoring the importance of health-related quality of life in the cost-effectiveness evaluation.

Increasing the baseline incidence of cervical cancer by 2.5  led to a decrease in the ICER for the 2-dose strategy, indicating a change in cost-effectiveness as disease prevalence shifts. Moreover, variations in vaccine dose pricing resulted in a range of approximately ±SAR 10 000/QALY from the base case, displaying sensitivity to these price changes. Conversely, the ICER showed limited sensitivity to variations in coverage and treatment costs, with narrower impacts of approximately ±SAR 5000/QALY, suggesting these factors affected cost-effectiveness less than the other parameters explored.

## DISCUSSION

This study utilized a compartmental deterministic dynamic transmission model to assess the uncertainty in health and cost-effectiveness outcomes given uncertainty in single-dose effectiveness and demonstrated that the 2-dose 9vHPV vaccination program likely significantly reduces the burden of HPV-related diseases compared with the 1-dose program. Key findings indicated that the 2-dose regimen of the 9vHPV vaccine could prevent approximately 13 700 additional cases of HPV-related diseases over its lifespan. Furthermore, the 2-dose program yielded a mean ICER of SAR 30 400 per QALY gained, suggesting favorable cost-effectiveness relative to a WTP threshold of approximately SAR 129 000 (equivalent to 1 GDP per capita).

The findings of this study indicated that a 2-dose 9vHPV vaccination regimen provides improved health outcomes by preventing a greater number of HPV-related disease cases and offers a more cost-effective approach than the 1-dose regimen. Specifically, for an initial cervical cancer incidence of 2.44 per 100 000 women, the model projected that the 2-dose program would lower the median cervical cancer incidence to 0.4 cases per 100 000 women, compared with 1.7 cases per 100 000 for the 1-dose program. Assuming a scenario analysis with higher cervical burden estimates than reported values (ie, cervical cancer incidence of 6.1/100 000 women), the model shows that the 2-dose program achieves the elimination threshold of 4 cases per 100 000 within 54 years, whereas the 1-dose program would take more than 100 years to achieve this elimination threshold (approximately twice as long). These results highlight the importance of adhering to the 2-dose recommendation to maximize the public health impact of HPV vaccination.

Moreover, scenario analyses that accounted for variations in cervical cancer incidence and vaccine effectiveness further support the findings regarding the benefits of the 2-dose program. This aligns with public health objectives outlined in KSA Vision 2030, which aims to enhance healthcare quality and promote preventive health measures.

### Limitations

While the findings provide valuable insights, several considerations should be mentioned. The model’s assumptions about HPV prevalence and vaccine effectiveness were based on specific studies, which may not fully capture local epidemiological dynamics, such as HPV co-infection. Additionally, variability in data quality, particularly relating to health utilities and vaccination coverage rates, may introduce some uncertainty in the projected outcomes. Sensitivity analysis was performed to address these uncertainties.

Another consideration is the lack of a national surveillance system for HPV-related diseases in KSA. This absence could affect the ability to conduct comprehensive epidemiological assessments, including accurate incidence and prevalence data, which are important for ensuring model accuracy. Further, the model’s reliance on long-term projections raises questions about the sustainability of vaccine effectiveness and the potential emergence of new HPV strains. Lastly, while the sensitivity analyses allowed for exploration of parameter variations, continued research would be beneficial to gather longitudinal data on vaccine efficacy and vaccination behaviors in the Saudi population.

Other limitations include the lack of robust long-term single-dose effectiveness data; there were no effectiveness data for males, no data on disease endpoints other than persistent infection, and no long-term duration of protection data. Therefore, we assumed the same waning and effectiveness proportions for all diseases as that which was available for studies available for cervical persistent infection. The actual waning functional form was not known but is usually assumed by most modelers and clinical scientists to reflect a decline in protection after some time. The choice of gamma distribution is nearly equivalent to assuming a normal distribution, which is typically used in agent-based models but is difficult to use in population-based models. Other assumptions could be a constant distribution, which would result in exponential loss of protection; this is widely not considered to be a reasonable form since it is not consistent with short-term trial results, which indicate no initial waning.

## CONCLUSION

This study presented evidence that the 2-dose 9vHPV vaccination regimen was more effective in terms of health outcomes and cost-effectiveness than the 1-dose strategy in KSA. By preventing a greater number of HPV-related diseases and demonstrating a favorable cost-effectiveness profile, the 2-dose program represents an important public health intervention to reach cervical cancer elimination. These findings underscore the need for targeted public health initiatives that advocate for the 2-dose regimen and enhance awareness of the benefits of vaccination, alongside efforts to improve cervical cancer screening programs in KSA.

As KSA progresses towards its Vision 2030 goals, adopting effective HPV vaccination strategies will be essential for mitigating the burden of HPV-related diseases and enhancing overall population health. Future research should prioritize refining model assumptions, monitoring long-term vaccine effectiveness, and assessing the real-world impact of these strategies to ensure optimal health outcomes in the region.

### Disclosures

V. Daniels and T.M. Malik are employees of Merck Sharp & Dohme LLC, a subsidiary of Merck & Co., Inc., Rahway, New Jersey, USA, a manufacturer of HPV vaccines, and may own stocks and/or stock options. C. Mamane and L. Lévy-Bachelot are employees of MSD France. D. Ugrekhelidze is an employee of MSD Switzerland. G. Albassam is an employee of MSD Saudi Arabia, and A.S. Bassyouni is an employee of MSD United Arab Emirates. A.M. Assiri and H.A.M. Alserehi are employees of the Ministry of Health in Saudi Arabia.

## Supplementary Material

Online Supplementary Material
