# Supplementary material for: Public Health Impact and Cost-Effectiveness of 2-Dose vs 1-Dose Human Papillomavirus Vaccination Regimen in Saudi Arabia
Source: J Health Econ Outcomes Res. 2026 Apr 23;13(1):130–9. doi: 10.36469/001c.160028 (PMC13110106; doi:10.36469/001c.160028)
Supplement: Online Supplementary Material [file jheor_2026_13_1_160028_341437.pdf]

Journal of Health Economics and Outcomes Research

Online Supplementary Material

Public Health Impact and Cost-Effectiveness of a 2-Dose vs 1-Dose HPV Regimen in Saudi Arabia. *JHEOR*. 2026;13(1):130-139. doi:10.36469/jheor.2026.160017

Model Inputs 2

Model Calibration 15

Deterministic Sensitivity Analysis 59

References 59

This supplementary material has been provided by the authors to give readers additional information about their work.

This is an open-access article distributed under the terms of the Creative Commons Attribution 4.0 International License (CCBY-4.0). View this license's legal deed at http://creativecommons.org/licenses/by/4.0 and legal code at http://creativecommons.org/licenses/by/4.0/legalcode for more information.

MODEL INPUTS

S1. Annual All-Cause Mortality Rates in the General KSA Population

Age group, years	Male	Female
0-1	0.006163935	0.005538731
1-4	0.000251218	0.000237235
5-9	0.000171512	0.000161966
10-14	0.000194361	0.000167373
15-19	0.000693824	0.000392084
20-24	0.001368659	0.000643042
25-29	0.001684273	0.000839536
30-34	0.002054307	0.001166414
35-39	0.002598967	0.001706103
40-44	0.003658811	0.002606425
45-49	0.005319581	0.004014730
50-54	0.007992640	0.006044989
55-59	0.011837314	0.008940912
60-64	0.017685077	0.013212230
65-69	0.025887300	0.019671940
70-74	0.038693306	0.031184991
75-79	0.059163846	0.050156156
80-84	0.091441375	0.082922034
≥85	0.177971281	0.173399479

Table values were obtained from life table data published by the World Health Organization [1].

S2. Sexual Behavior Factors

Percentages refer to the percent of the KSA population in the sexual behavior category. Mean values represent the mean number of partners among individuals in the sexual behavior category. Values for males were based the findings of a survey conducted among 225 male students in KSA, aged 15 to 20 years old [2].

Percent of the population in each of the following sexual activity categories

Sexual activity category	Value males [a]	Source
**Low (mean number of sexual partners per year: 0-1)**	69.0%	Raheel et al. 2013 [2]
**Medium (mean number of sexual partners per year: >2)**	31.0%	

[a] For male data we leverage Raheel et al. 2013 reporting 31% having had premarital sex.[2] For lack of any other data we assume these are in the high sexual activity group of 2+ and the rest are in the low group.

Values for females were based the findings of survey by Alhamlan et al. 2016 conducted among 400 women, aged 22 to 80 years old (mean age, 41 years), attending routine clinical care in KSA; only women who were or had ever been married were included in the survey [3].

Mean number of sexual partners per year by activity category for females

Number of sexual partners per year	Value females	Assume 10% for 0*	Model inputs	Source
**0**	-	10	89.6	Alhamlan et al. 2016 [3]
**1**	88.4%	79.6		
**2**	7.5%	6.8	10.4	
**>3**	4.0%	3.6		

*Alhamlan et al. 2016 provides 1, 2, and >=3. Since there is no data on number with 0 partners, we had to assume a percentage [3].

Mean number of sexual partners per year by activity category and gender: final inputs

Sexual activity category	Value males (number)	Value females (number)	Source
**Low (mean number of sexual partners per year: 0-1)**	0.94*	0.89*	Males: Assumptions Females: Alhamlan et al. 2016 [3]
**Medium (mean number of sexual partners per year: >2)**	5.0	3.0*	

* Calculations and assumptions as follows:
Females:
For group 0-1, we estimated the average number of lifetime partners from the weighted average of the 0 and 1 group in Alhamlan et al. 2016.[3]
For group 2+, we simply assumed the average from Alhamlan et al. 2016.

Males:
For group 0-1, we estimated the average of female value (0.89) and 1
For group 2+, we simply assumed 5.0

The above data on sexual categories are summarized in the following table:

**Sexual behavior category**	**Male**		**Female**	
	**%**	**Mean value**	**%**	**Mean value**
Mean number of sexual partners, per year				
Low: 0-1	69.00%	0.94	89.6%	0.89
Medium: ≥2	31.00%	5.0	10.4%	3.00

In the model, sexual mixing among members of different age cohorts was represented by a parameter ranging from 0 (minimal mixing) to 1 (maximal mixing).

How much sexual mixing is there among members of different age cohorts?

**Degree of randomness for ages**	**Mixing**	**Source**
**Between debut and cessation**	0.5	Assumption
**After cessation**	0.9	Assuming older age groups are more random

How much sexual mixing is there among members of different sexual activity groups?

	**Mixing**	**Source**
**Degree of randomness for sexual activity groups**	0.6	Assume slight bias toward random

S3. Mean Number of Sexual Partners by Age Group and Gender, KSA

Age group, years	Males, n	Females, n
<15	0	0
15-17	0.05	0.01
18-20	0.13	0.03
21-25	0.17	0.05
26-29	0.18	0.06
30-34	0.16	0.06
35-39	0.14	0.05
40-44	0.12	0.05
45-49	0.1	0.05
50-54	0.09	0.04
55-59	0.08	0.04
60-64	0.07	0.04
65-69	0.06	0.03
70-74	0.05	0.03
75-79	0.05	0.03
80-84	0.04	0.03
≥85	0.03	0.02

KSA, the Kingdom of Saudi Arabia

Table data reflect findings of a published survey conducted in KSA [3] from which parameters for the current study were derived. The model assumed that the mean lifetime number of partners followed a lognormal age distribution, with sexual debut occurring at the age of 15 years. For females, the mean cumulative lifetime number of partners was assumed to be 1.2, a value reached at the age of 41 years. For males, the mean cumulative lifetime number of partners was assumed to be 3.0, a value reached at the age of 35 years.

S4. HPV Genotype Attribution in HPV-Related Disease Cases

Disease	HPV 6	HPV 11	HPV16	HPV18	HPV31	HPV33	HPV45	HPV52	HPV58	Source
Cervical cancer			60.00	11.00	3.00	3.00	6.00	4.00	4.00	de Sanjose et al. 2010 [4] *
Vaginal cancer			59.00	5.00	5.00	5.00	4.00	3.00	4.00	Alemany et al. 2014 [5] ‡
Vulvar cancer			68.00	4.60	1.30	5.90	2.90	1.80	1.10	Serrano et al. 2015 [6] ‡
Anal cancer, female			83.40	3.60	1.80	3.10	0.70	0.30	2.00	Serrano et al. 2015 [6] ‡
Anal cancer, male			80.70	3.60	1.90	2.70	0.90	0.70	1.80	Alemany et al. 2015 [7] ‡
Penile cancer			68.70	1.50	0.80	2.40	2.70	1.20	1.30	Alemany et al. 2016 [8] ‡
Oropharyngeal cancer, female			20.70	0.45	0	0.82	0.10	0	0.17	Castellsague et al. 2016 [9] #
Oropharyngeal cancer, male			20.70	0.45	0	0.82	0.10	0	0.17	Castellsague et al. 2016 [9] #
Genital warts	81.00	9.00								Saraiya 2015

Table values represent the percentages.
* HPV type attribution for Asia
‡ HPV type attribution for world population
# Separate data for male and female oropharyngeal cancer attribution were not available, we used world-wide data. We used the type distribution over HPV-positive oropharyngeal cancers worldwide.

Table S5. HPV-Related Disease Patterns: Annual Mortality Rates Associated with Cervical, Vaginal, Vulvar, Anal, Head and Neck and Penile Cancer and Per Disease Stage (Local, Regional, Distant)

	Local	Regional	Distant	Source
**Cervical cancer**				
15-39 years	0.031332	0.070298	0.427872	Alkhalawi et al. 2022 [10]
40-54 years	0.023846	0.058338	0.387357	
55-64 years	0.027336	0.085538	0.502487	
65-74 years	0.136855	0.198553	0.45739	
≥75 years	0.38654	0.557792	0.437462	
**Vaginal cancer**				
15-29 years	0.04	0.226	0.428	US Surveillance, Epidemiology, and End Results (SEER) [11]
30-39 years	0.04	0.226	0.428	
40-49 years	0.022	0.105	0.428	
50-59 years	0.097	0.087	0.428	
60-69 years	0.054	0.174	0.428	
≥70 years	0.221	0.297	0.428	
**Vulvar cancer**, women aged ≥15 years	0.0468	0.1834	0.581	National Health Service of the United Kingdom (NHS) [12]
**Anal cancer**				
Women aged ≥15 years	0.042269	0.148165	0.555	National Health Service of the United Kingdom (NHS) [12]
Men aged ≥15 years	0.033048	0.08666	0.37	
**Head & neck cancer** [a]				
<57 years, both genders	0.065092	0.155457	0.318895	Alsbeih et al. 2019 [13]
≥57 years, both genders	0.127503	0.286537	0.573735	
**Penile cancer**				
15-54 years	0.069	0.136	0.436	US Surveillance, Epidemiology, and End Results (SEER) [11]
55-64 years	0.055	0.142	0.33	
≥65 years	0.098	0.182	0.584	

[a] Oropharyngeal cancer estimates in KSA from Alsbeih et al. 2019: derived from survival probabilities in Figure 2A of the publication. The resulting annual probability of dying in one year is the average of the probability of dying in years 1 through 4. Alsbeih et al. determined that differences by gender were not significant [13].

Table S6. Screening Parameters and Vaccination Coverage Rates, KSA

	Values	Source(s)
**Cervical screening rates**		
Percent of females ever screened (age 25-65 years)	15%	ICO/IARC KSA fact sheet [14]
Annual probability of being screened in females aged 25-65 years (among those that are ever screened)	2.35%	ICO/IARC KSA fact sheet [14] Using the average annual rate estimated from the 25-65 years old screened every 3 years and every 5 years and assuming a constant rate
Screening follow-up	72.5%	Assuming 72.5% receive follow-up after a positive screen [15]
**Diagnostic performance data on Pap screening and colposcopy**		
Cervical disease		
Cytology specificity	0.94	Bigras et al 2005 [16] and Coste et al. 2003 [17]
Colposcopy sensitivity	0.96	Mitchell et al. 1998 [18]
Colposcopy specificity	0.48	Mitchell et al. 1998 [18]
Cytology sensitivity for CIN1	0.28	Bigras et al 2005 [16]
Cytology sensitivity for CIN2	0.59	Bigras et al 2005 [16]
Cytology sensitivity for CIN3+	0.59	Bigras et al 2005 [16]
**HPV vaccination coverage rates** [a]		
Year 2022		
11-12-year-olds	52.0%	Data on file*
13-14-year-olds	52.0%	Data on file*
Year 2023		
11-12-year-olds	63.0%	Data on file*
13-14-year-olds	63.0%	Data on file*

CIN, cervical intraepithelial neoplasia; CIS, carcinoma *in situ*; HPV, human papillomavirus; KSA, the Kingdom of Saudi Arabia

[a] MSD data on file. Projected vaccination coverage rate of 76% was assumed to be reached by year 2028.

*MoH data, consistent with WHO estimations: https://immunizationdata.who.int/global/wiise-detail-page/human-papillomavirus-(hpv)-vaccination-coverage?CODE=SAU&YEAR=

Table S7. Cervical Cancer Disease Pattern: Hysterectomy Rates

**Women receiving hysterectomy for cervical cancer per year** [a]		
Age group, years	**Value (percent)**	**Source**
15-29	0.200	Kumari et al. 2022 [19]
30-39	3.300	
40-49	9.700	
≥50	9.700	

[a] Estimates for hysterectomy were based on a National Family Health Survey from India (2015-2016) [19]

**Age group, years**	**Rate per 1,000 women** [a]
0-14	0
15-17	0.212
18-20	0.212
21-25	0.212
26-29	0.212
30-34	4.109
35-39	4.109
40-44	7.997
45-49	7.997
50-54	7.197
55-59	3.598
60-64	2.399
65-69	1.799
70-74	1.439
75-79	1.199
80-84	1.028
≥85	0.9

[a] The data used for estimating hysterectomy annual incidence rate was derived from self-reported hysterectomy prevalence in Kumari 2022 [19]. Annual incident rates were estimated through a simple two-state model for those with and without hysterectomy in a population with constant size and age distribution.

Table S8. Costs Associated with HPV, Screening Procedures and HPV-Related Diseases (Stratified by Sex and Disease Severity)

	HPV-related costs	
	Vaccine costs	
**HPV vaccination per dose**		
9vHPV	605.00	
Administration cost	15.38	
	Screening costs	
	**Male**	**Female**
**Screening procedure**		
Screening (PAP smear)	-	511.00
Colposcopy	-	635.00
Biopsy	-	1,062.00
	HPV-related diseases costs per episode of care	
	**Male**	**Female**
**HPV-related disease**		
Cervix		
CIN 1	-	3,169.70
CIN 2	-	3,169.70
CIN 3, CIS	-	3,169.70
Local cancer	-	75,963.00
Regional cancer	-	89,852.90
Distant cancer	-	96,145.90
Vagina		
VaIN 1	-	1,737.00
VaIN 2	-	1,737.00
VaIN 3, CIS	-	1,737.00
Local cancer	-	46,318.90
Regional cancer	-	45,776.10
Distant cancer	-	68,726.70
Vulva		
Local cancer	-	53,438.60
Regional cancer	-	84,597.90
Distant cancer	-	76,515.80
Cancer survivor	-	-

	**HPV-related costs**	
Penile cancer		
Local disease	92,034.70	-
Regional disease	49,225.90	-
Distant disease	48,758.20	-
Anal cancer		
Local disease	36,786.90	36,786.90
Regional disease	38,487.70	38,487.70
Distant disease	90,598.20	90,598.20
Cancer survivor		
Head & Neck cancer		
Local disease	50,749.50	50,749.50
Regional disease	92,708.80	92,708.80
Distant disease	59,722.20	59,722.20
Cancer survivor		
Genital warts	9,252.30	9,252.30
Recurrent respiratory papillomatosis	12,566.60	12,566.60

9vHPV, 9-valent HPV vaccine; CIN, cervical intraepithelial neoplasia; CIS, carcinoma *in situ*; HPV, human papillomavirus; KSA, the Kingdom of Saudi Arabia; VaIN, vaginal intraepithelial neoplasia; QALY, quality-adjusted life year

Calculated costs were based on interviews with 12 key opinion leaders in KSA [20]. All costs given in the Saudi riyal (SAR). Screening costs were inclusive of office visit. In the model, costs were discounted at an annual rate of 3%. Disease stages relate to the traditional Tumor-Node-Metastasis (TNM) classification system as follows: ‘Local disease’ corresponds to TNM stages I and II (i.e., localized primary tumor), ‘Regional disease’ to TNM stage III (i.e., metastasis to regional lymph nodes), and 'Distant disease’ to TNM stage IV (i.e., distant metastatic disease).

S9. Health Utilities

Age-specific health utility values in the healthy population were based on previously published EQ-5D scores [21].

Age-specific health utility values in the healthy population

Age group, years	Male	Female
18-24	0.935	0.914
25-34	0.921	0.904
35-44	0.900	0.877
45-54	0.864	0.846
55-64	0.842	0.812
65-74	0.825	0.803
≥75	0.773	0.741

EQ-5D, EuroQol-5 Dimension; QALY, quality-adjusted life year
Utility values refer to the QALY, which has a value between 0 and 1. In the model, QALYs were discounted at an annual rate of 3%.

Health utility values for cancer patients were derived from various sources. The table below summarizes the health utility values used to estimate QALYs relating to HPV-related diseases in the population.

Utilities in population with HPV-related diseases

Condition	Gender	Value [-20%, +20%]	Source
CIN1	Female	0.91 [0.73, 1.00]	Insinga et al., 2007 [22]
CIN 2/3, VaIN 2/3, CIS	Female	0.87 [0.7, 1.00]	Insinga et al., 2007 [22]
Local cervical/vaginal/vulvar cancer	Female	0.76 [0.61, 0.91]	Myers et al., 2004 [23]
Regional cervical/vaginal/vulvar cancer	Female	0.67 [0.54, 0.80]	Myers et al., 2004 [23]
Distant cervical/vaginal/vulvar cancer	Female	0.48 [0.38, 0.58]	Gold et al., 1998 [24]
Cervical/vaginal/vulvar cancer survivor	Female	0.76 [0.61, 0.91]	Wenzel et al., 2005 [25] and assumption
Local anal cancer	Female, Male	0.76 [0.61, 0.91]	Myers et al., 2004 [23]
Regional anal cancer	Female, Male	0.67 [0.54, 0.80]	Myers et al., 2004 [23]
Distant anal cancer	Female, Male	0.48 [0.38, 0.58]	Gold et al., 1998 [24]

Condition	Gender	Value [-20%, +20%]	Source
Anal cancer survivor	Female, Male	0.76 [0.61, 0.91]	Wenzel et al., 2005 [25] and assumption
Local penile cancer,	Male	0.76 [0.61, 0.91]	Myers et al., 2004 [23]
Regional penile cancer	Male	0.67 [0.54, 0.80]	Myers et al., 2004 [23]
Distant penile cancer	Male	0.48 [0.38, 0.58]	Gold et al., 1998 [24]
Penile cancer survivor	Male	0.76 [0.61, 0.91]	Wenzel et al., 2005 [25] [23]and assumption
Local H&N cancer	Female, Male	0.76 [0.61, 0.91]	Myers et al., 2004 [23]
Regional H&N cancer	Female, Male	0.67 [0.54, 0.80]	Myers et al., 2004 [23]
Distant H&N cancer	Female, Male	0.48 [0.38, 0.58]	Gold et al., 1998 [24]
H&N cancer survivor	Female, Male	0.76 [0.61, 0.91]	Wenzel et al., 2005 [25] and assumption
Genital warts	Female, Male	0.91 [0.73, 1.00]	Myers et al., 2004 [23]
RRP	Female, Male	0.796 [0.64, 0.96]	Lindman et al., 2005 [26]

S10. Calibration targets for incidence of human papillomavirus-related cancers per 100,000 population in the calibrated model, the Kingdom of Saudi Arabia

		**Overall**	**25-29**	**30-34**	**35-39**	**40-44**	**45-49**	**50-54**	**55-59**	**60-64**	**65-69**	**70-74**	**75-79**	**80-84**	**≥85**
M	Genital warts	0.89	0.89	0.89	0.89	0.89	0.89	0.89	0.89	0.89	0.89	0.89	0.89	0.89	0.89
F	Genital warts	0.89	0.89	0.89	0.89	0.89	0.89	0.89	0.89	0.89	0.89	0.89	0.89	0.89	0.89
F	Cervical	2.44	0.29	1.62	3.23	4.67	6.09	6.92	7.74	9.07	10.40	12.00	13.10	14.30	14.40
F	Vaginal	0.03	0.00	0.00	0.00	0.00	0.10	0.00	0.23	0.35	0.00	0.70	0.00	0.00	0.00
F	Vulvar	0.10	0.00	0.07	0.00	0.08	0.21	0.16	0.23	0.35	0.42	1.41	2.02	1.78	2.88
M	Penile	0.02	0.00	0.00	0.00	0.00	0.00	0.08	0.11	0.00	0.32	0.66	0.00	0.00	0.00
F	Anal	0.20	--	0.07	--	0.08	0.10	0.33	0.94	1.39	2.09	2.82	3.02	3.57	5.75
M	Anal	0.27	0.06	0.25	0.18	0.04	0.48	0.78	0.78	0.94	1.59	1.99	1.97	1.99	3.50
F	Oral cavity	1.35	0.29	0.28	0.83	1.40	2.48	3.63	4.69	5.58	8.77	13.40	17.10	19.60	20.10
M	Oral cavity	1.26	0.18	0.45	0.72	1.05	1.45	2.26	3.45	6.39	8.88	9.27	13.80	21.90	31.50
F	Oropharynx	0.05	0.00	0.00	0.08	0.16	0.10	0.00	0.23	0.00	0.00	0.70	1.01	0.00	0.00
M	Oropharynx	0.06	0.00	0.05	0.00	0.00	0.00	0.16	0.45	0.19	0.32	0.66	0.98	1.99	0.00
F	Larynx	0.13	0.00	0.00	0.08	0.00	0.21	0.33	1.17	0.35	1.25	1.41	1.01	1.78	2.88
M	Larynx	0.89	0.00	0.00	0.18	0.44	0.86	2.26	3.67	5.26	8.56	9.27	8.86	9.97	14.00

F, female; M, male

The estimated incidence of oral cavity cancer among girls was 0 for those aged 0 to 14 years, 0.9 for those aged 15 to 19 years, and 0.27 for those aged 20 to 24 years. The estimated incidence of oral cavity cancer among boys was 0.07 for those aged 0 to 4 years, 0.0 for those aged 5 to 19 years, and 0.33 for those aged 20 to 24 years.

Inputs used in the calibrated incidence model were based on multiple sources, including findings of a genotyping study of 285 patients with head and neck cancers in KSA (2002-2016) [13] and cancer incidence data from the International Agency for Cancer Research (IARC) [27].

S11. Calibration targets for mortality from human papillomavirus-related cancers per 100,000 population in the calibrated model, the Kingdom of Saudi Arabia

		Overall	25-29	30-34	35-39	40-44	45-49	50-54	55-59	60-64	65-69	70-74	75-79	80-84	≥85
F	Cervical	1.22	0.07	0.28	1.05	1.79	2.58	3.46	4.69	5.93	7.52	9.15	11.10	12.50	14.40
F	Vaginal	0.01	--	--	--	--	--	--	0.23	--	0.42	--	--	--	--
F	Vulvar	0.03	--	--	--	--	0.10	--	--	--	--	0.70	1.01	1.78	2.88
M	Penile	0.01	--	--	--	--	--	0.08	0.11	--	--	--	--	--	--
F	Anal	0.07	--	--	--	--	--	0.16	0.47	0.70	0.84	1.41	1.01	1.78	
M	Anal	0.10	--	--	--	0.04	0.21	0.47	0.45	0.38	0.63	0.66	0.98	--	--
F	Oral cavity	0.62	0.15	--	0.45	0.55	0.93	1.65	2.35	3.14	4.60	6.34	8.06	8.92	11.50
M	Oral cavity	0.51	0.12	0.25	0.09	0.44	0.43	0.86	1.89	3.01	3.81	3.97	4.92	7.98	14.00
F	Oropharynx	0.04	--	--	--	0.16	0.10	--	0.23	--	--	0.10	1.01	--	--
M	Oropharynx	0.04	--	--	--	--	--	0.08	0.33	0.19	0.32	0.66	0.98	1.99	--
F	Larynx	0.08	--	--	--	--	0.10	0.16	0.70	0.35	0.84	0.70	1.01	1.78	2.88
M	Larynx	0.46	--	--	0.05	0.13	0.21	0.78	2.00	3.57	5.08	5.30	4.92	7.98	14.00

F, female; M, male

The estimated mortality from oral cavity cancer among girls aged 15 to 19 years was 0.09. Inputs used in the mortality model were based multiple sources, including cancer survival data from the National Health Service of the United Kingdom (NHS)[12] and the International Agency for Cancer Research (IARC) [27], and an analysis of cervical cancer survival based on data from the Saudi Cancer Registry (SCR) [10].

MODEL CALIBRATION

The HPV model is a collection of models consisting of one model for each of the 7 high risk HPV vaccine types (16, 18, 31, 33, 45,52, and 58) for the cervical, vaginal, vulvar, penile, anal, and oropharyngeal sites and two models for the low-risk HPV types (6, 11) combining genital warts, RRP, and low grade CIN. Each of these 44 models is calibrated separately. The calibration process consists of estimating certain model parameters (depending on the site and type being calibrated) by minimizing an objective function involving the calibration target data and the corresponding model output. For all calibrations we minimize a residual function which is the weighted sum of squared differences between the equilibrium model outcome and the target outcome data. The following sections show the resulting model fit to cancer incidence (Table S10) and mortality (Table S11) data along with applied attribution (Table S4).

Anal

Fit for HPV16 cancer and mortality incidence

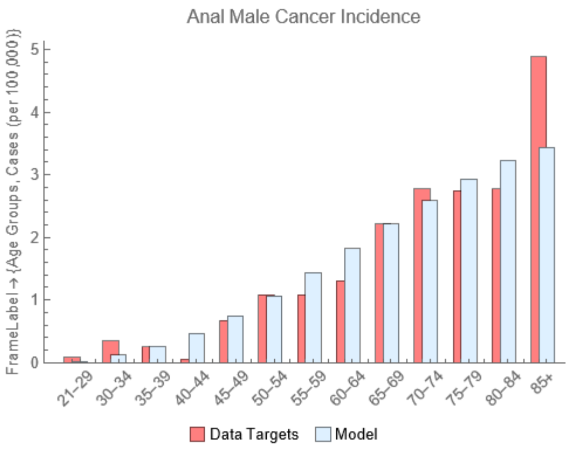

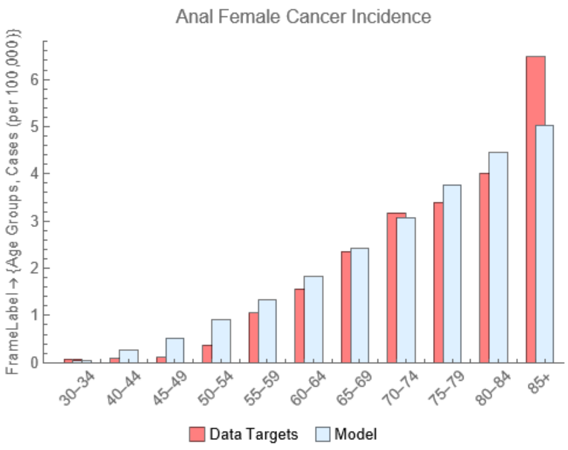

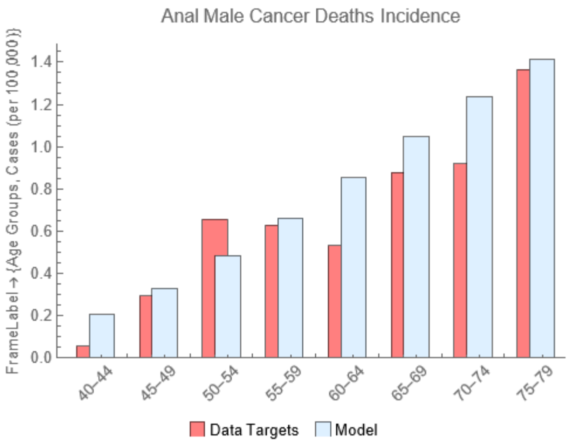

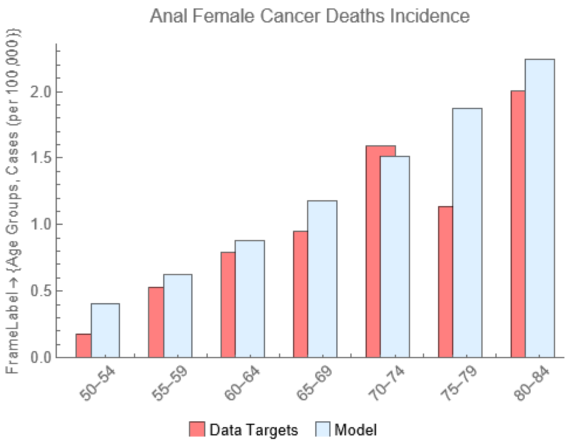

Fit for HPV18 cancer and mortality incidence

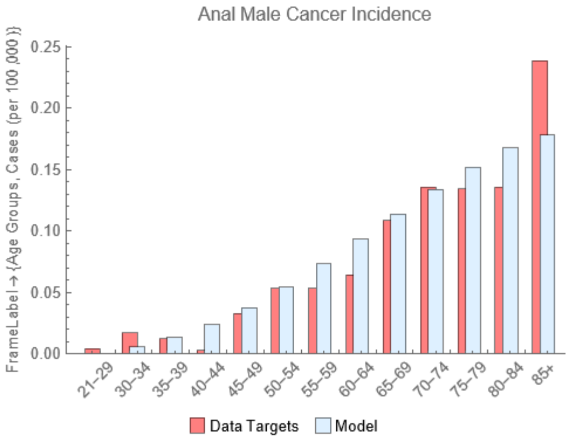

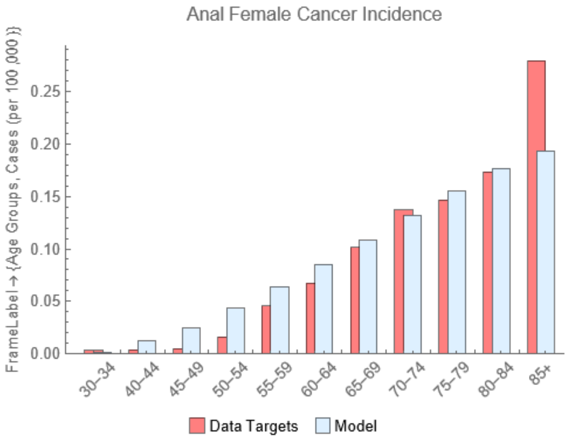

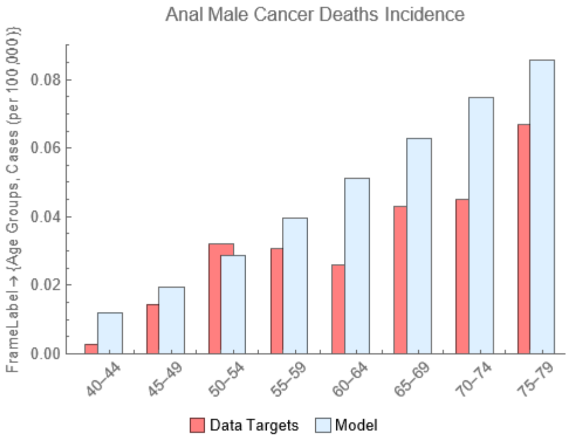

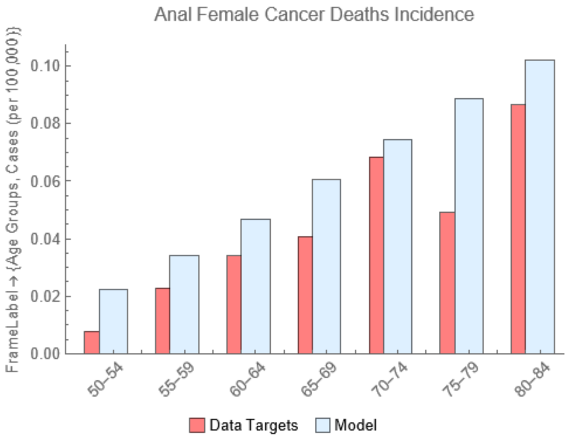

Fit for HPV31 cancer and mortality incidence

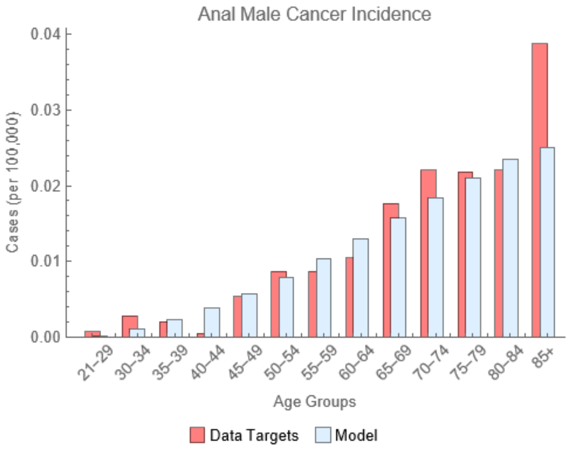

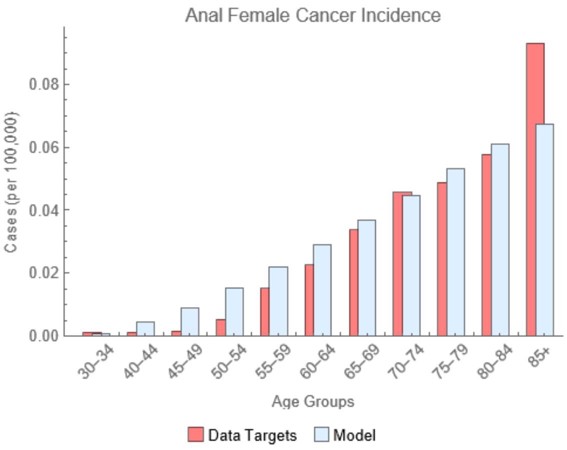

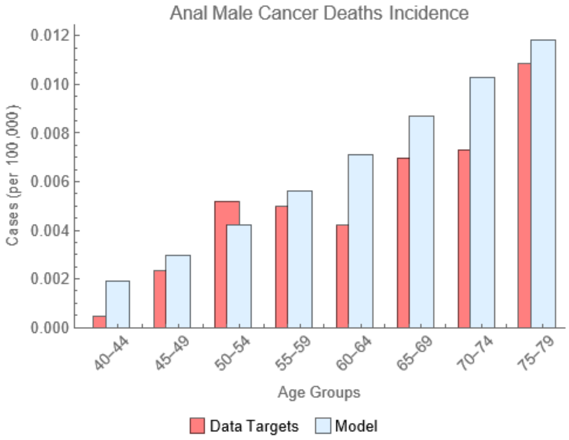

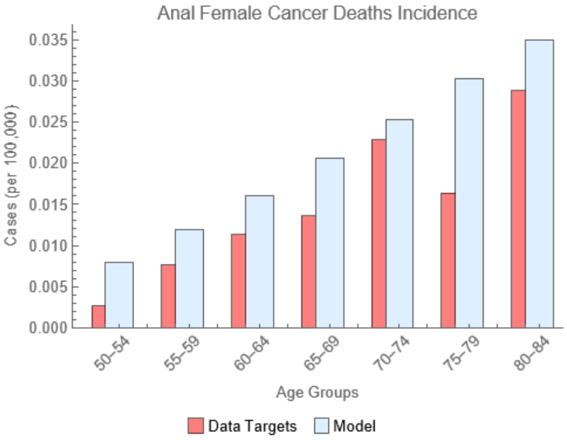

Fit for HPV33 cancer and mortality incidence

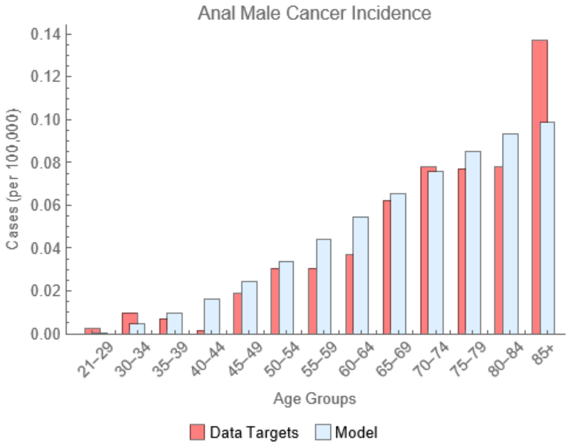

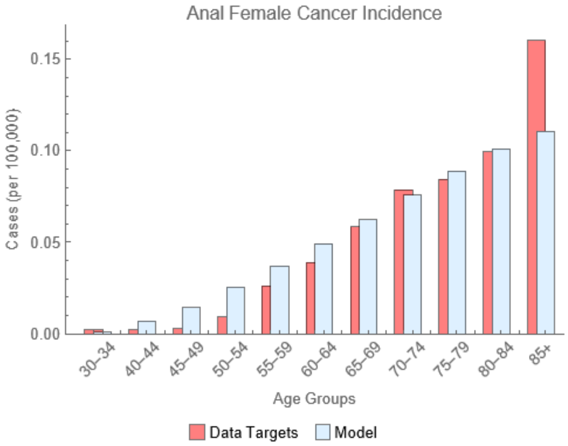

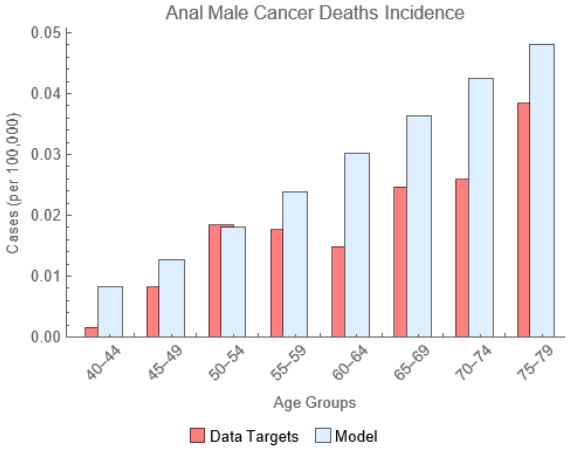

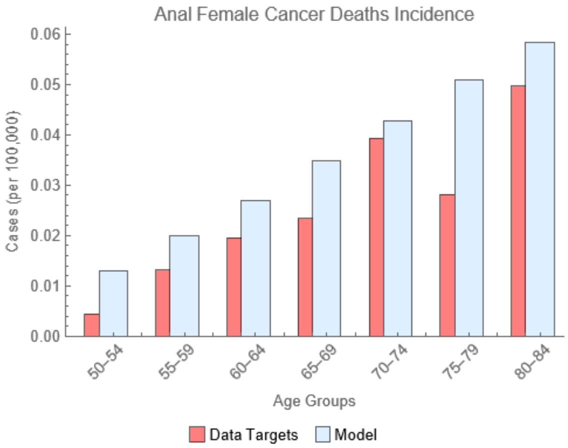

Fit for HPV45 cancer and mortality incidence

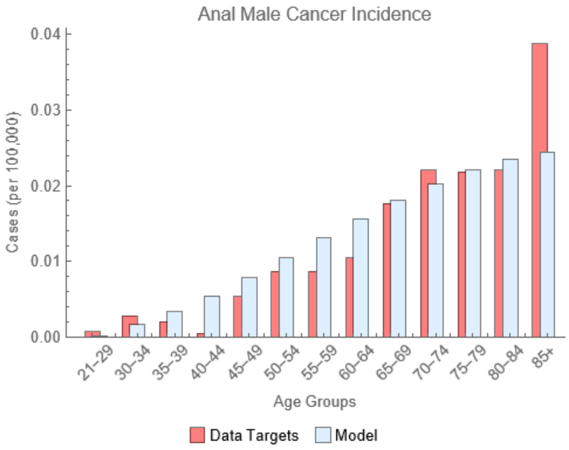

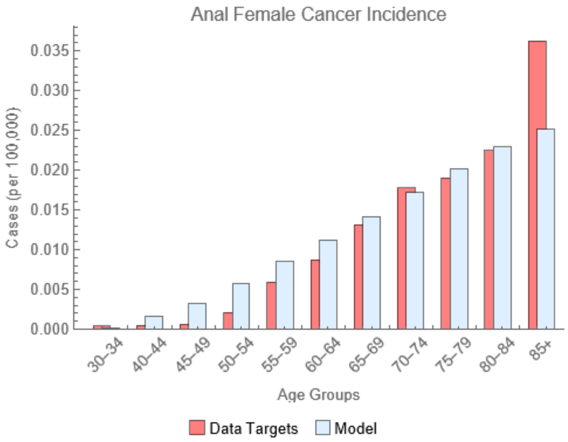

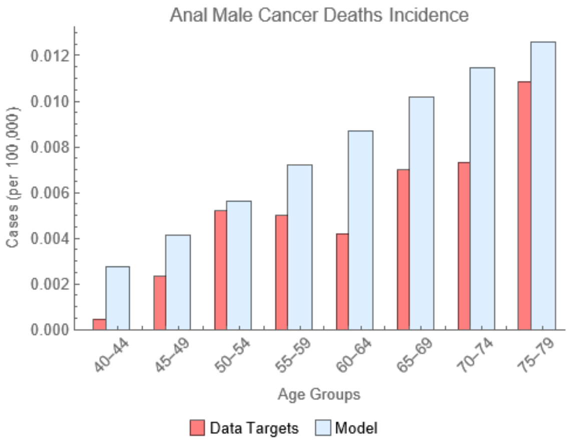

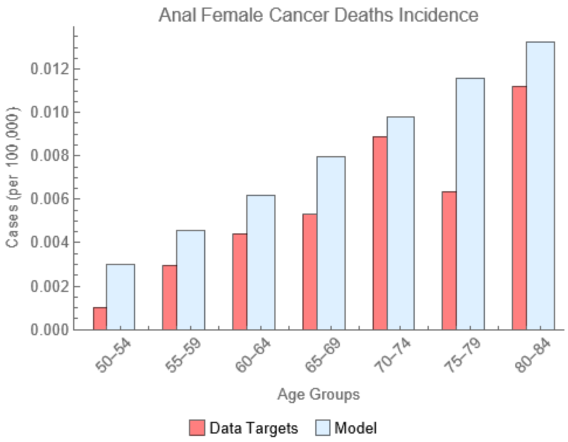

Fit for HPV52 cancer and mortality incidence

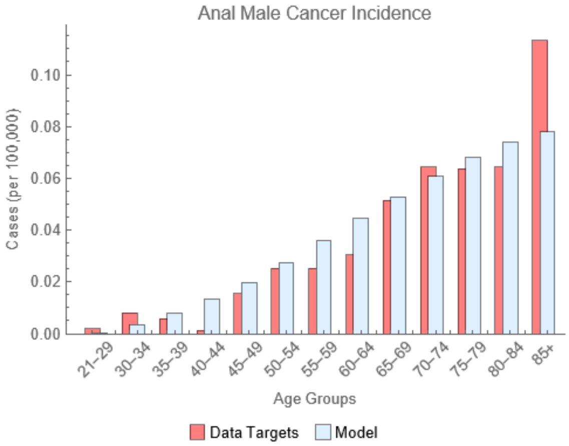

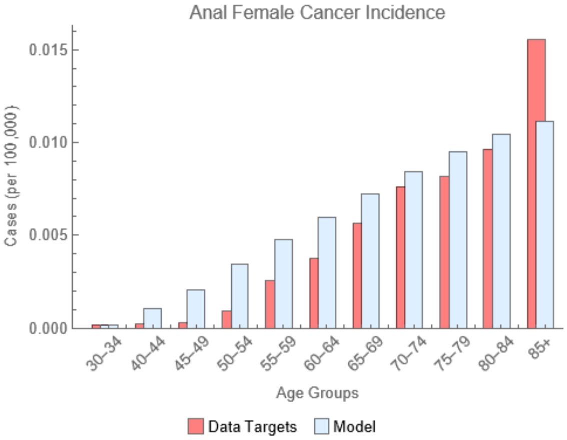

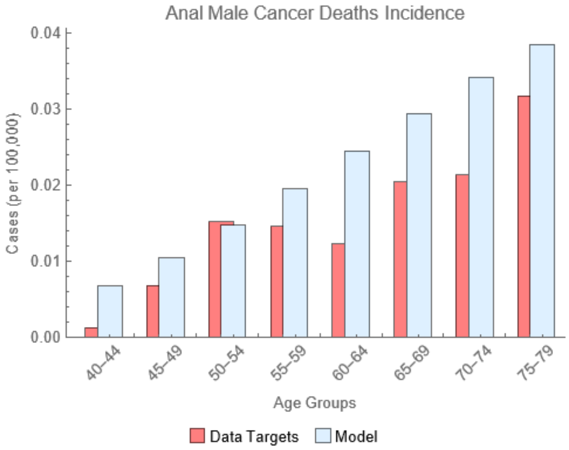

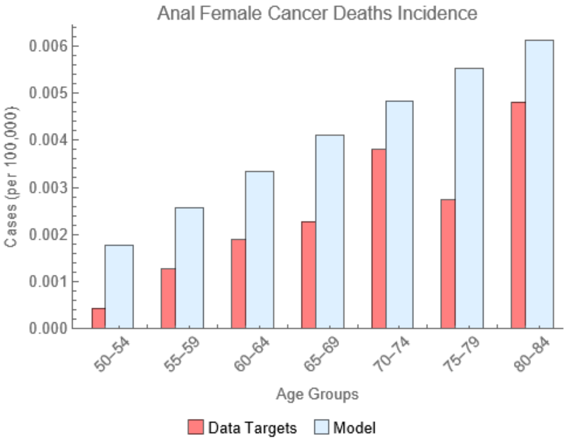

Fit for HPV58 cancer and mortality incidence

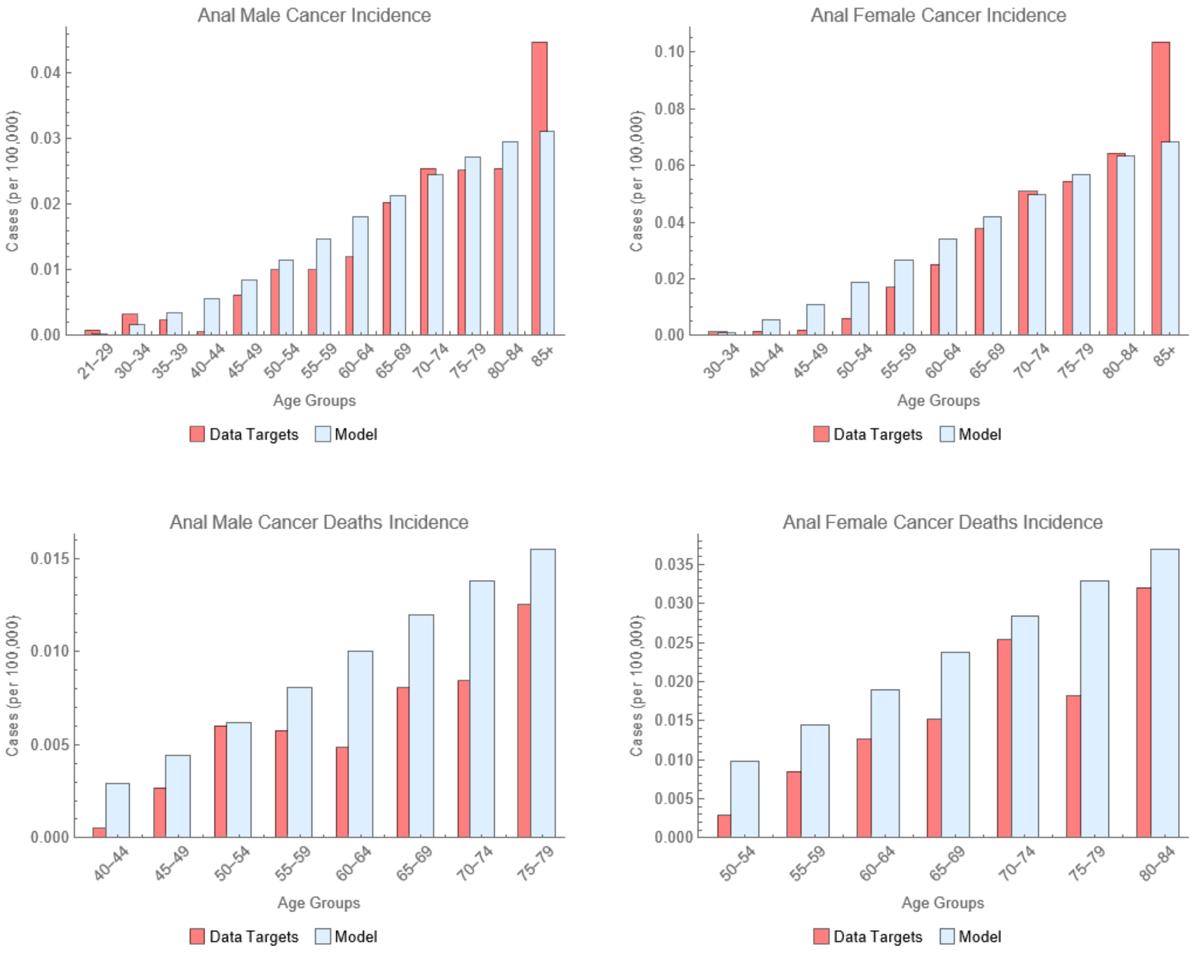

Cervical

Fit for HPV16 cancer, mortality incidence, and genital prevalence

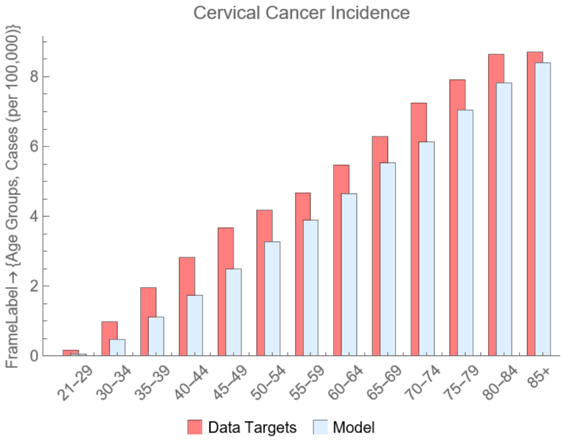

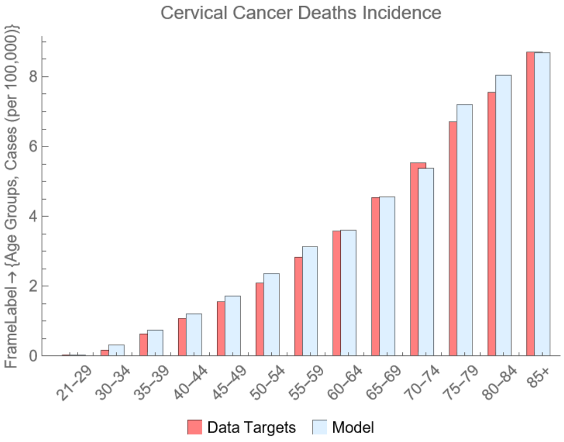

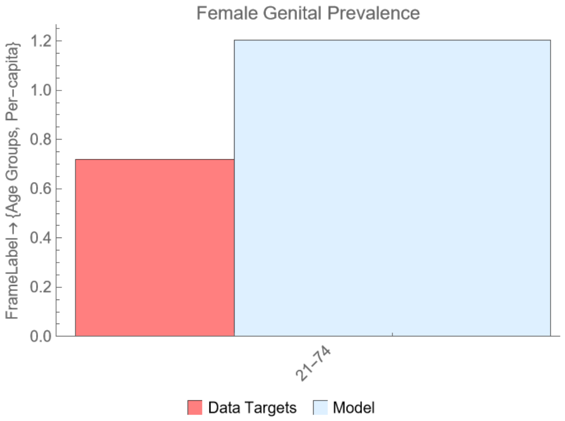

Fit for HPV18 cancer and mortality incidence and genital prevalence

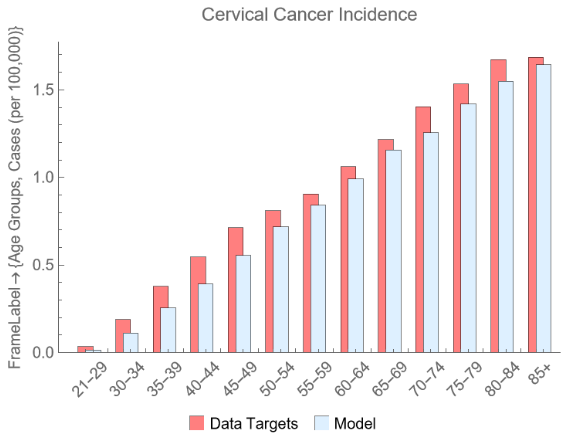

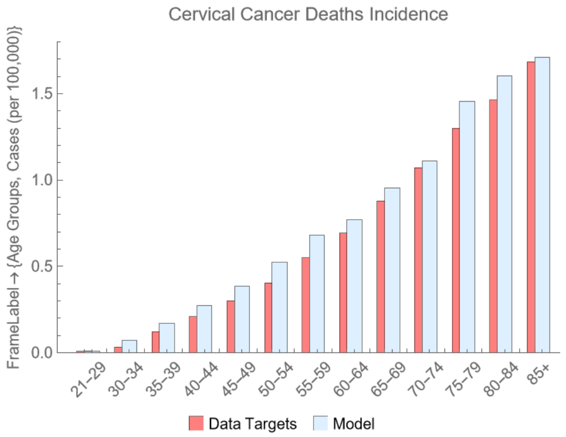

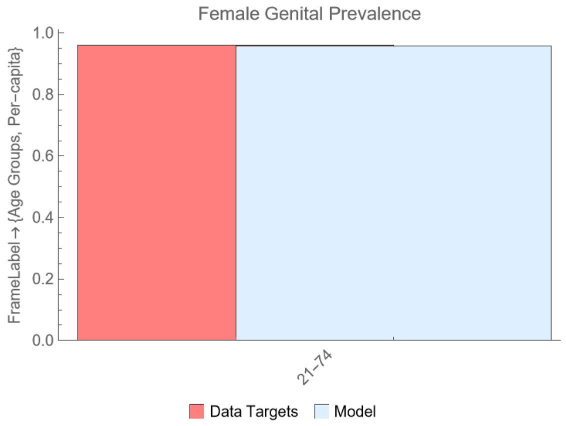

Fit for HPV31 cancer and mortality incidence and genital prevalence

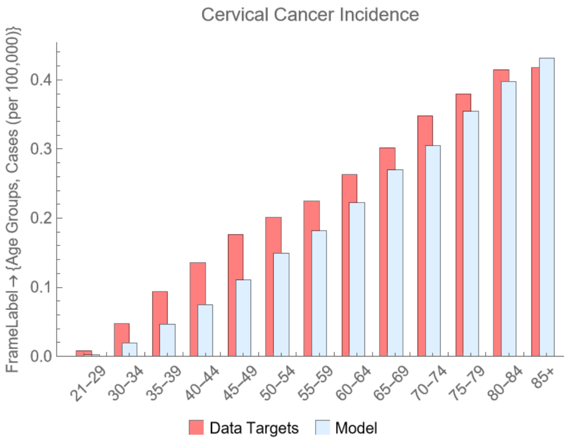

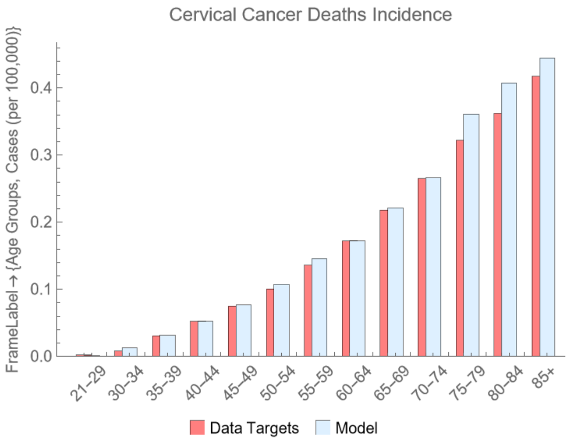

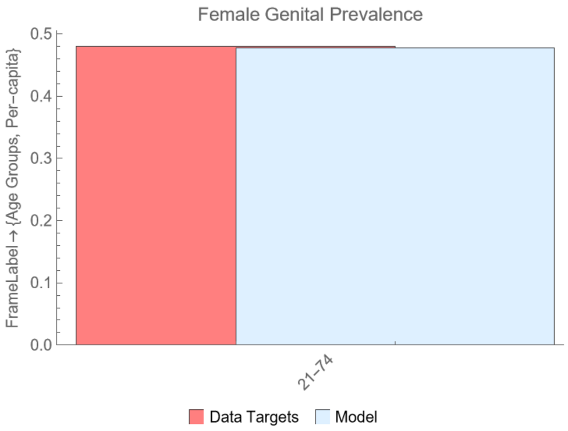

Fit for HPV33 cancer and mortality incidence and genital prevalence

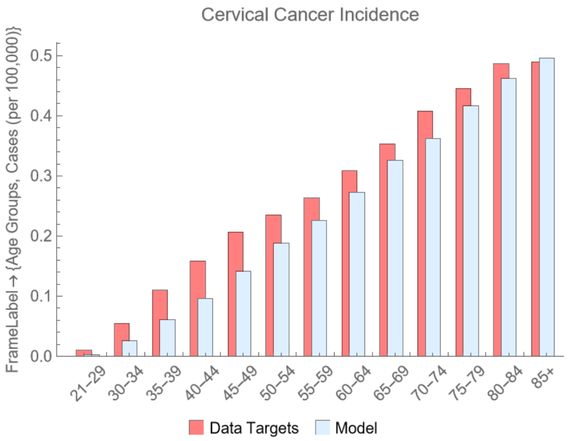

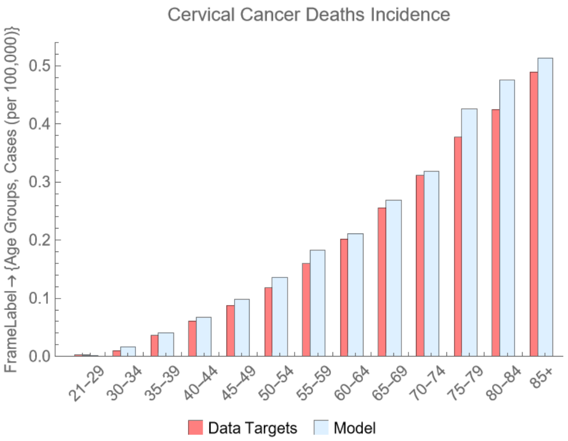

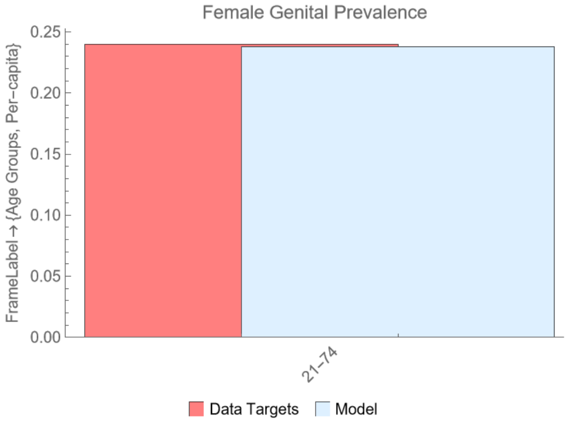

Fit for HPV45 cancer and mortality incidence and genital prevalence

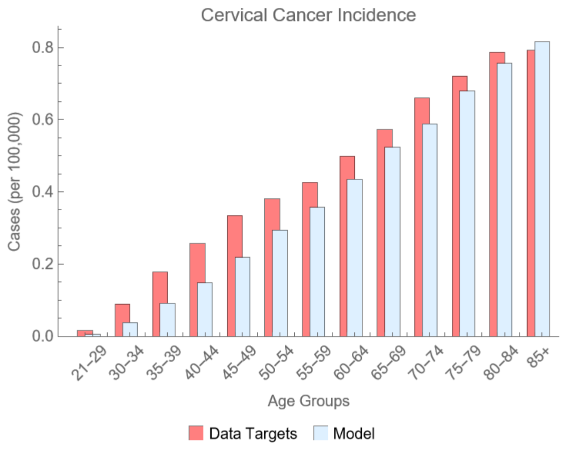

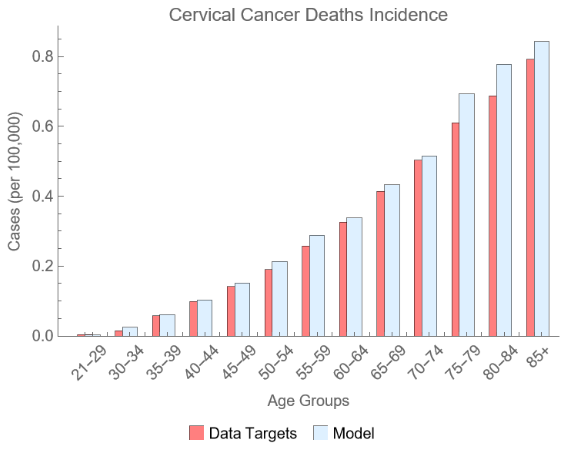

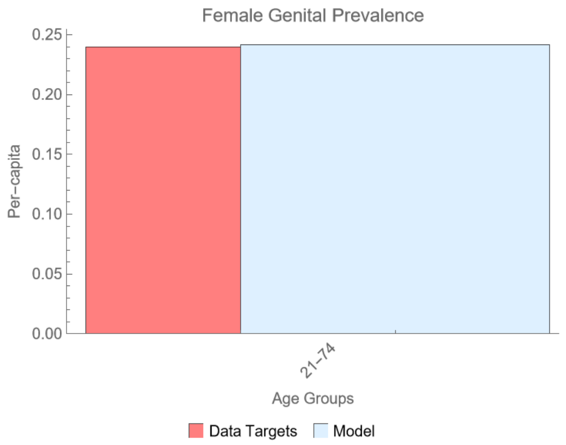

Fit for HPV52 cancer and mortality incidence and genital prevalence

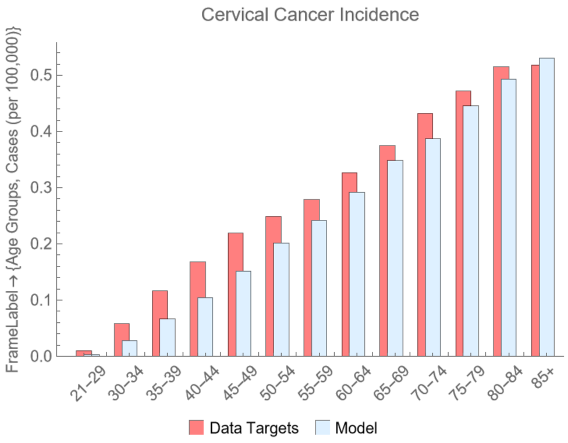

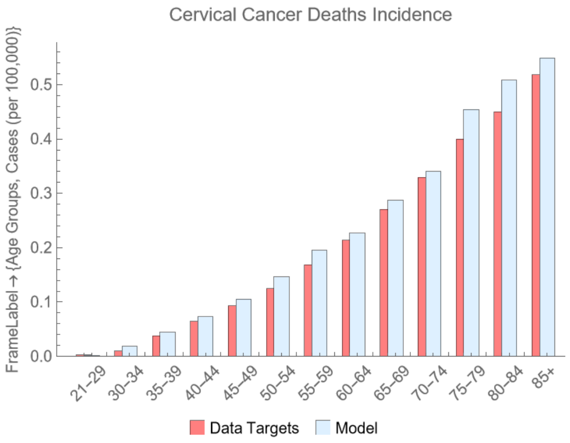

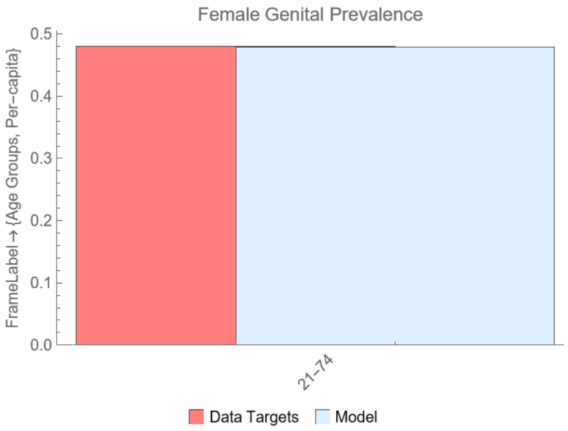

Fit for HPV58 cancer and mortality incidence and genital prevalence

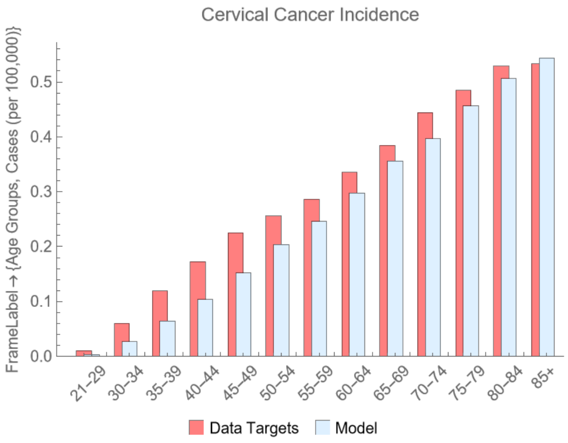

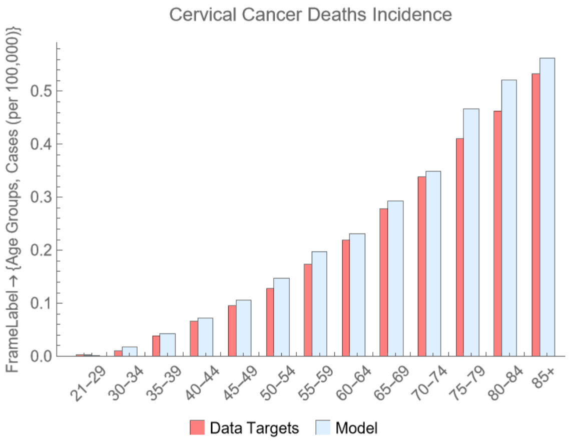

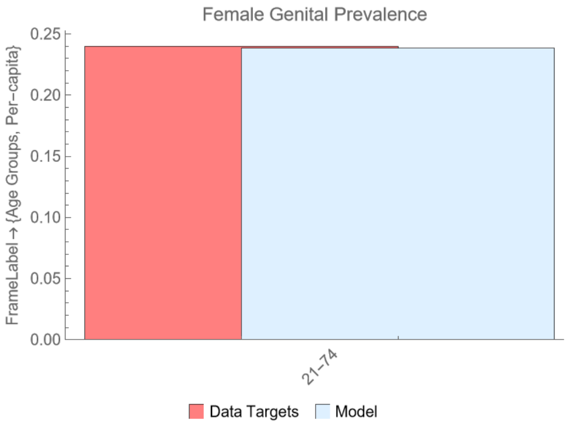

Genital warts

Fit for HPV6 genital warts incidence

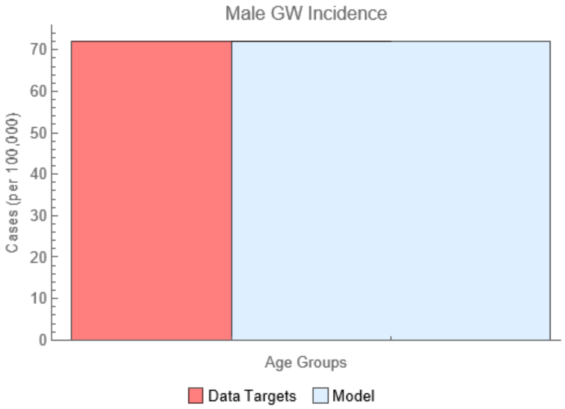

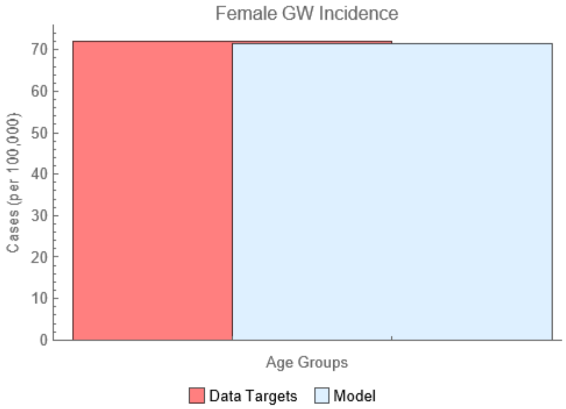

Fit for HPV11 genital warts incidence

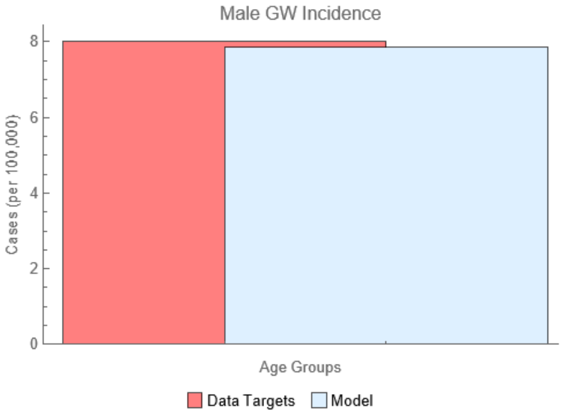

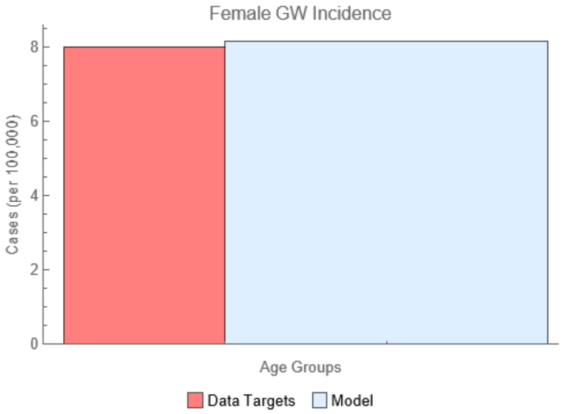

Head and Neck

Fit for HPV 16 cancer and mortality incidence

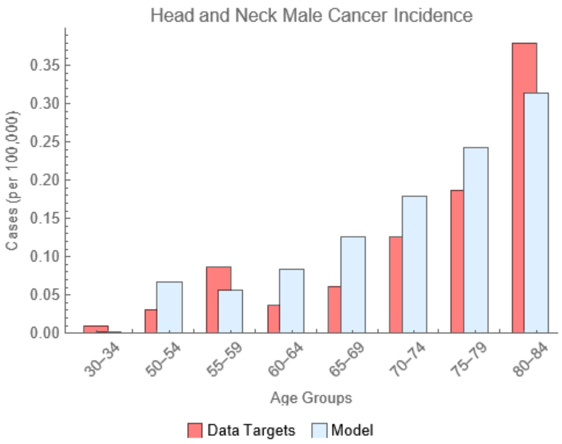

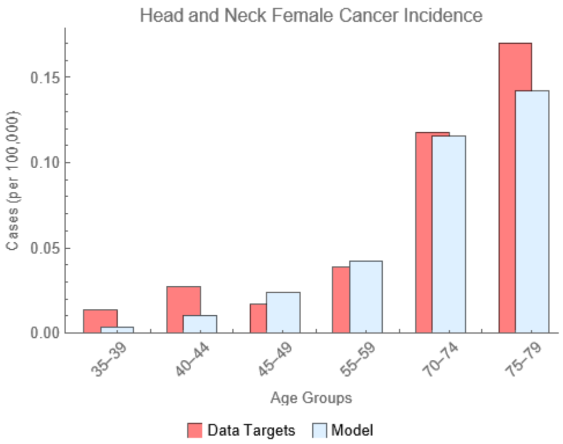

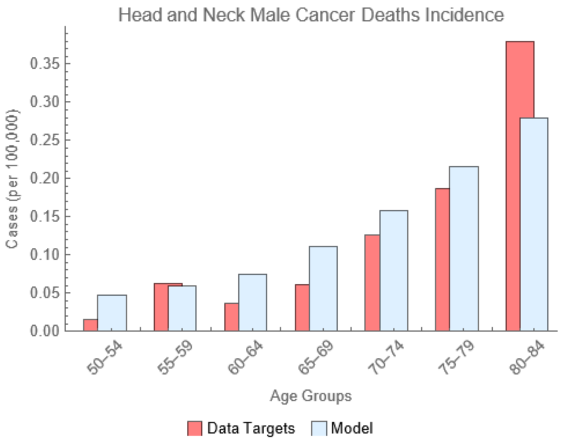

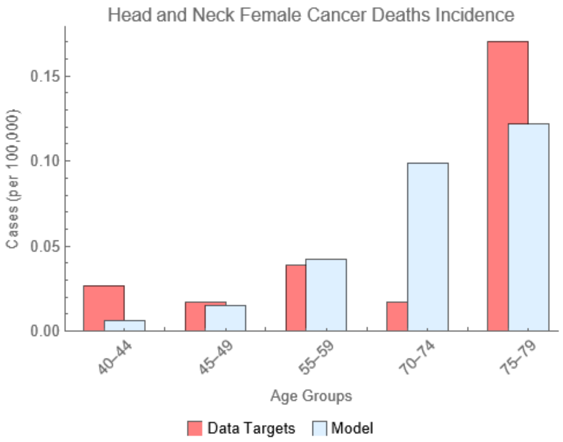

Fit for HPV18 cancer and mortality incidence

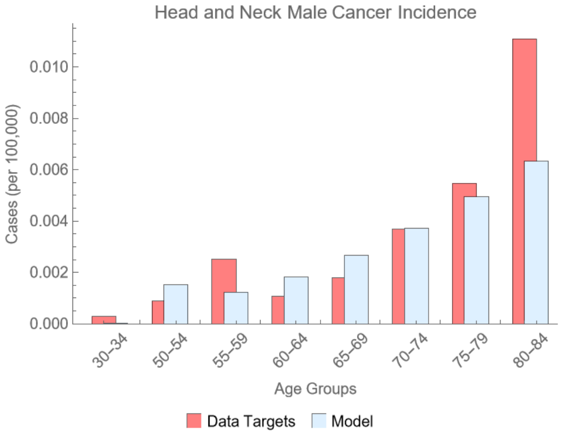

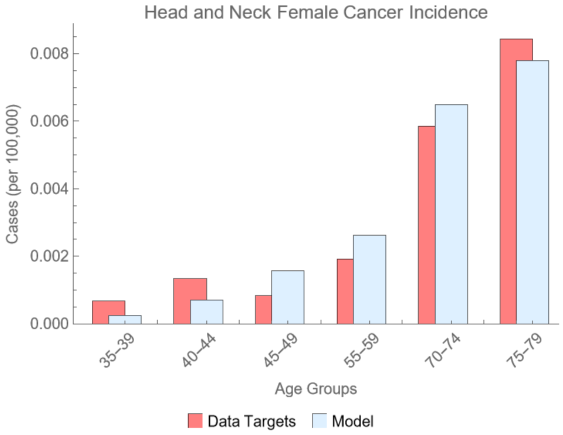

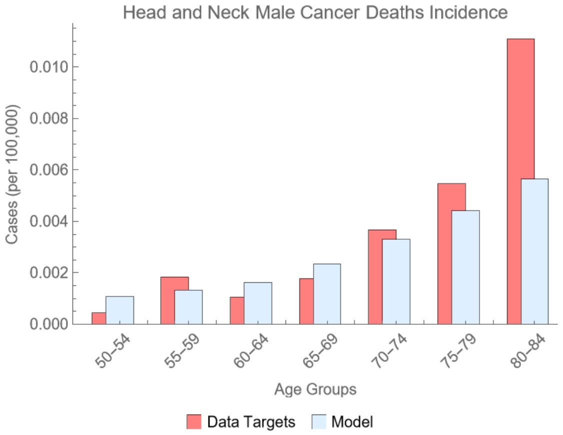

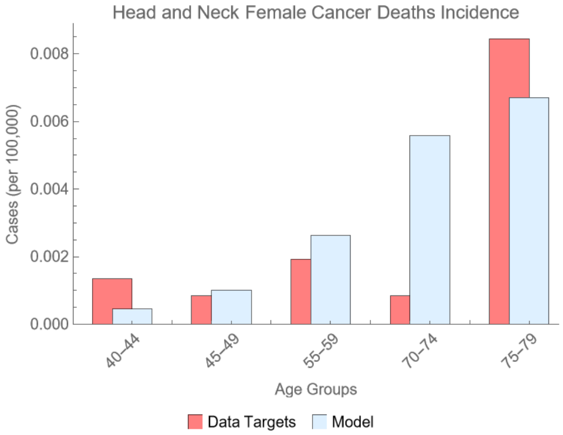

Fit for HPV31 cancer and mortality incidence

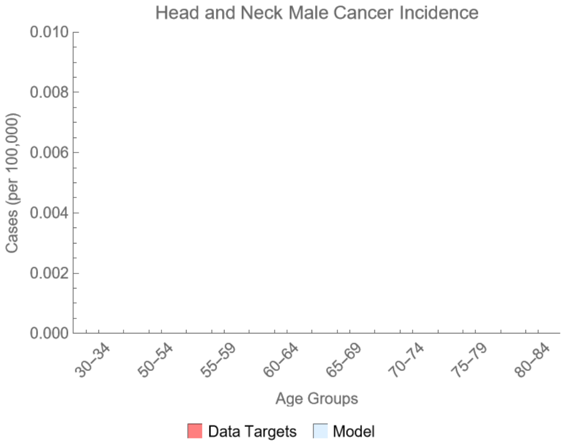

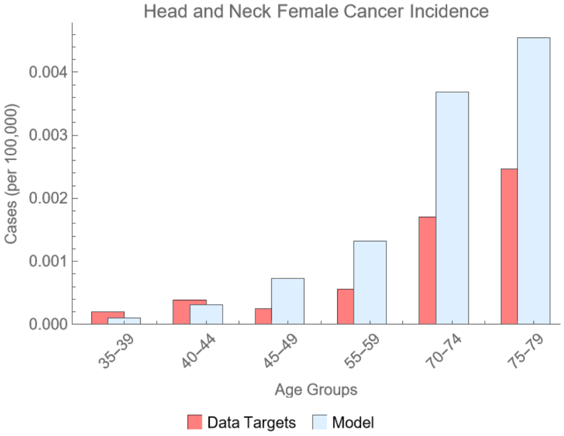

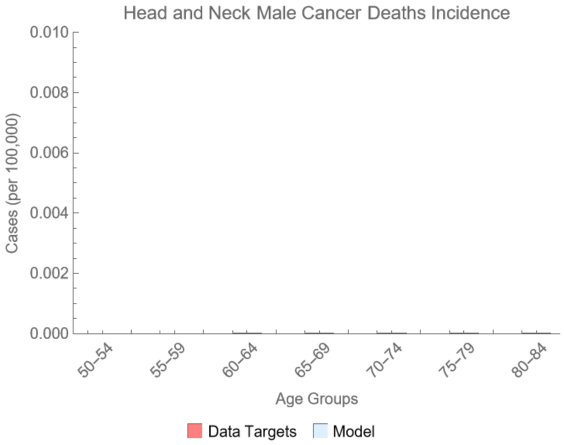

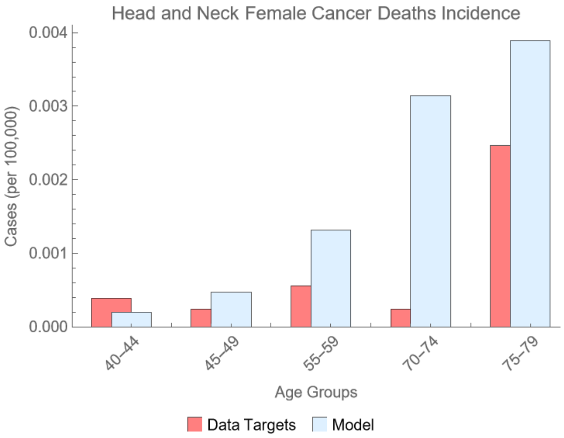

Note, there is no observed male head and neck cancer attribution for HPV31

Fit for HPV33 cancer and mortality incidence

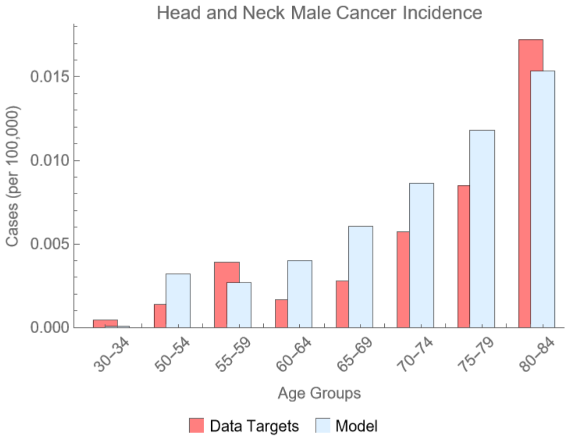

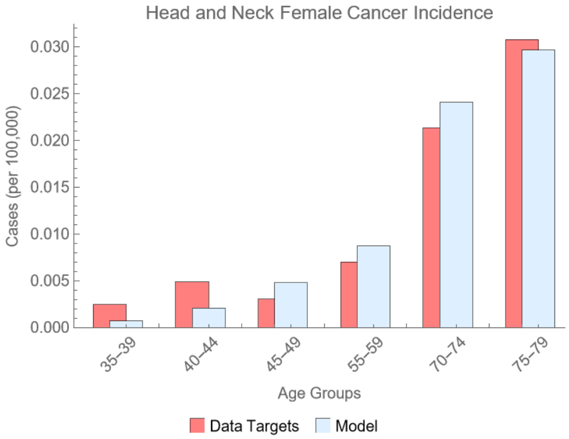

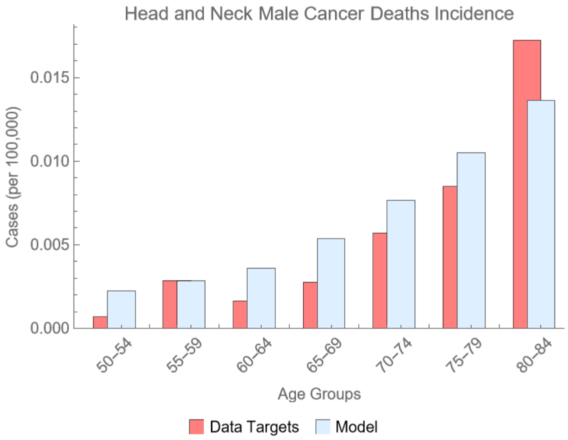

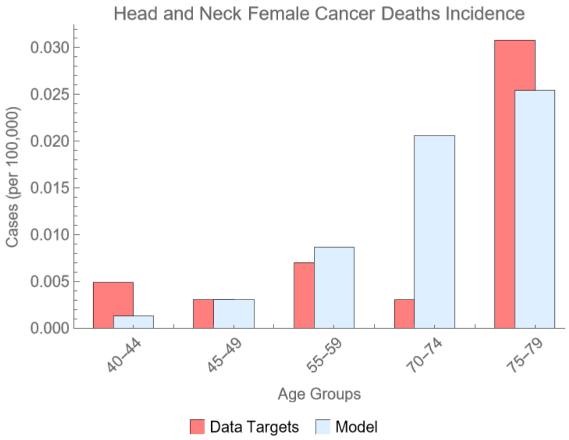

Fit for HPV45 cancer and mortality incidence

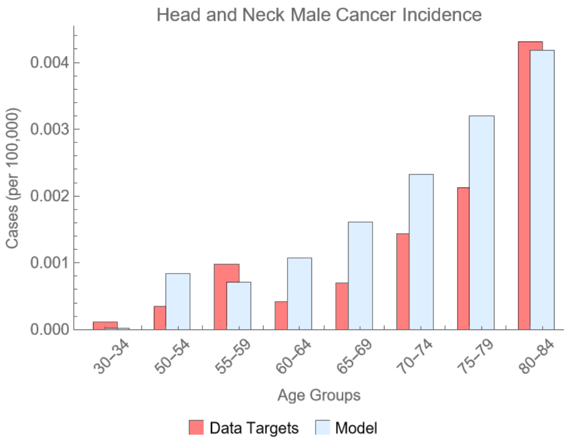

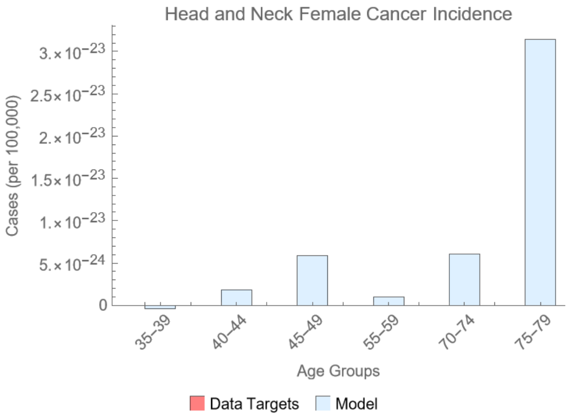

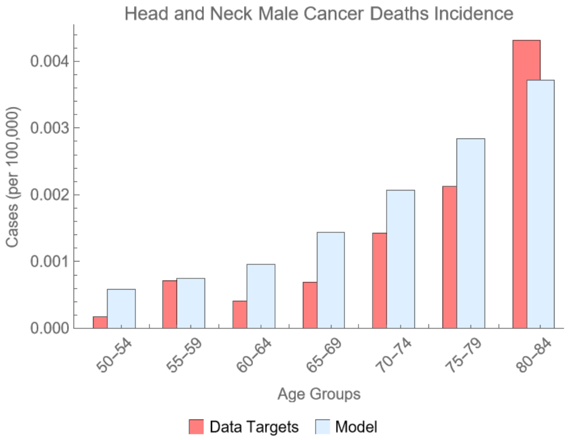

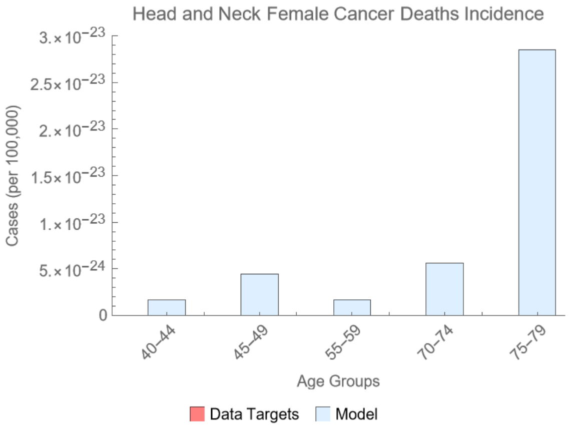

Note there is no observed attribution for HPV45 female incidence

Fit for HPV52 cancer and mortality incidence

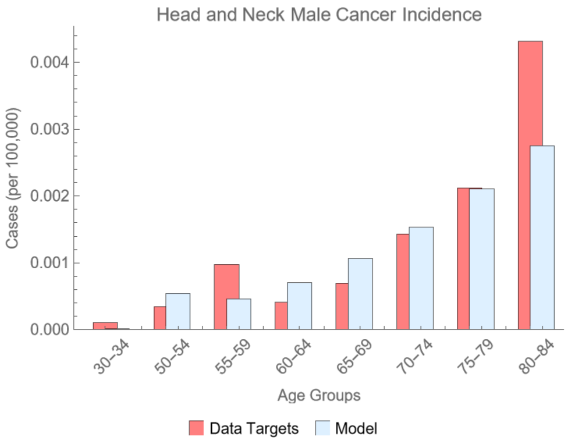

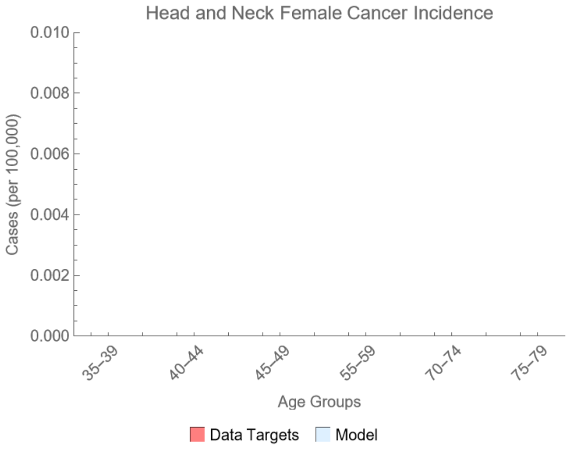

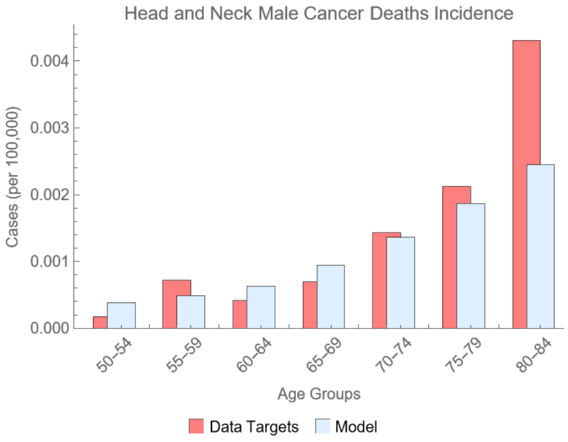

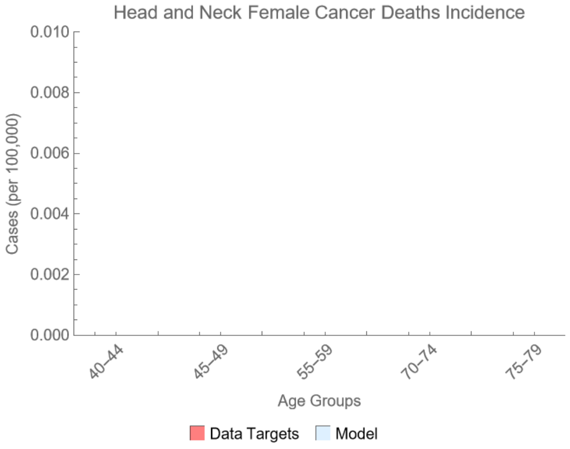

Note there is no observed attribution for female head and neck HPV52 attribution

Fit for HPV58 cancer and mortality incidence

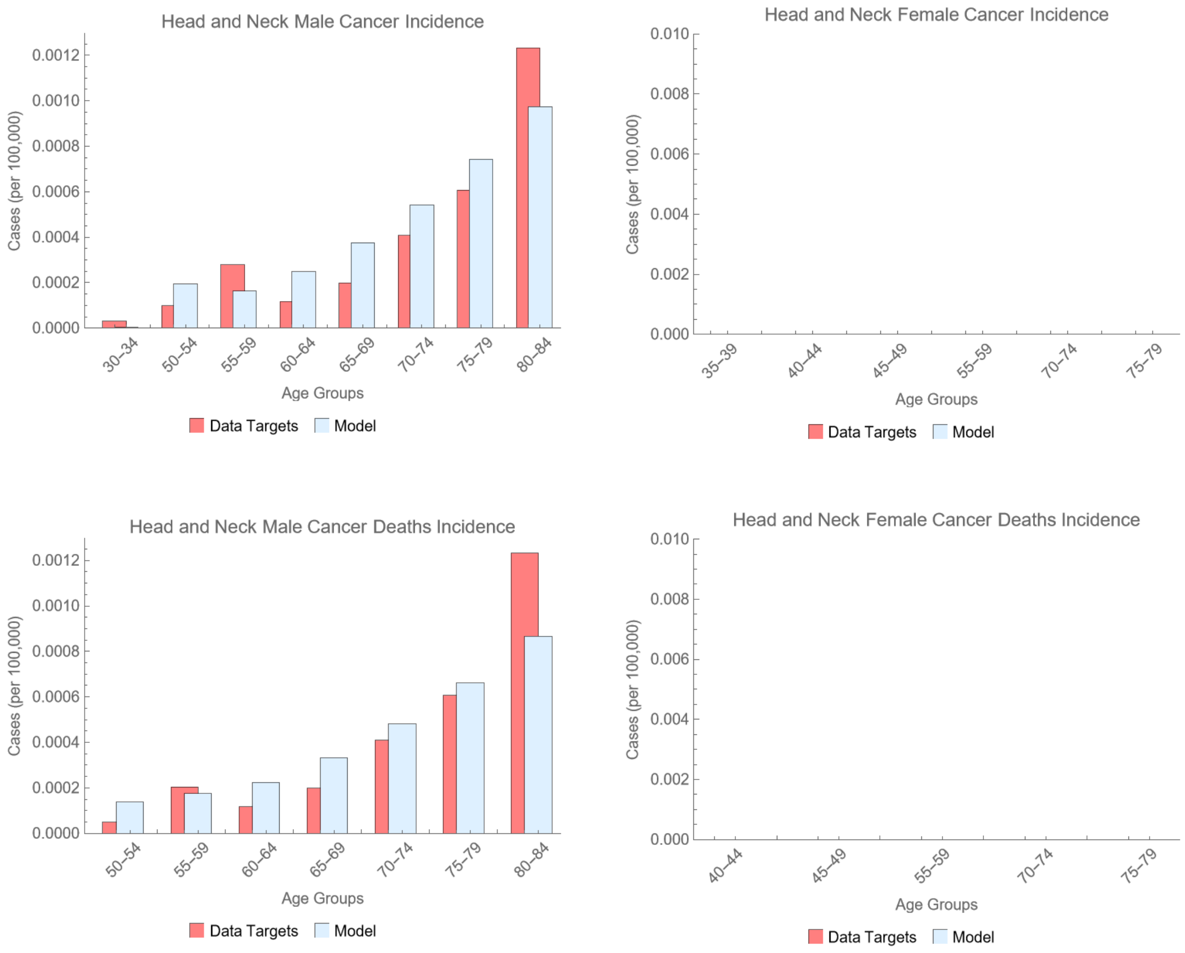

Note there is no observed attribution for female head and neck HPV58 attribution

Penile

Fit for HPV 16 cancer and mortality incidence and female genital prevalence

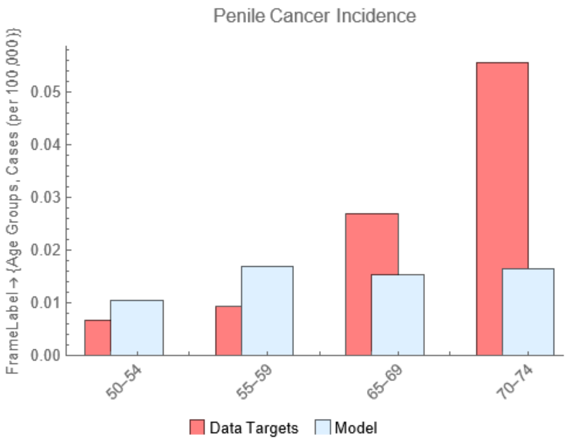

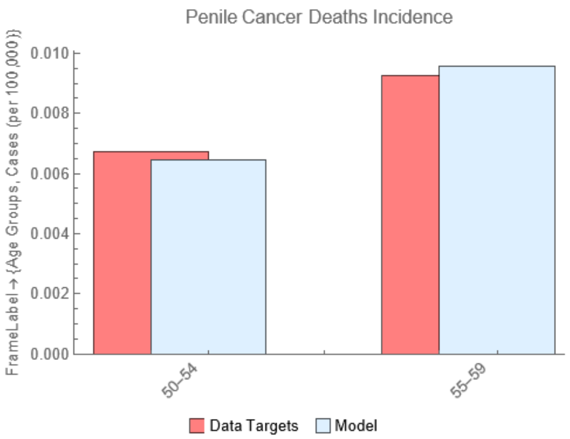

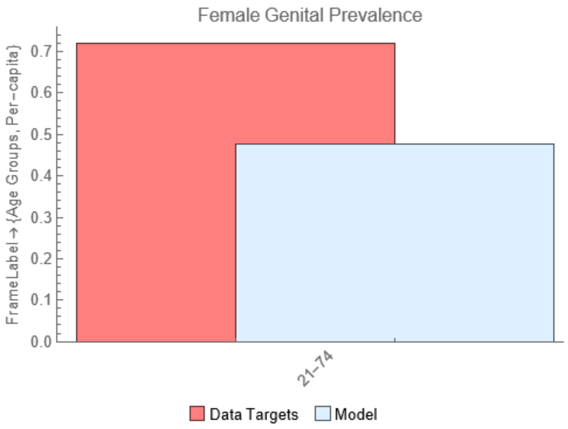

Fit for HPV18 cancer and mortality incidence and female genital prevalence

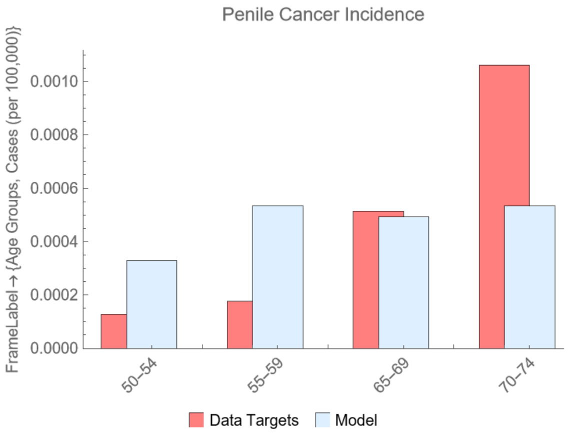

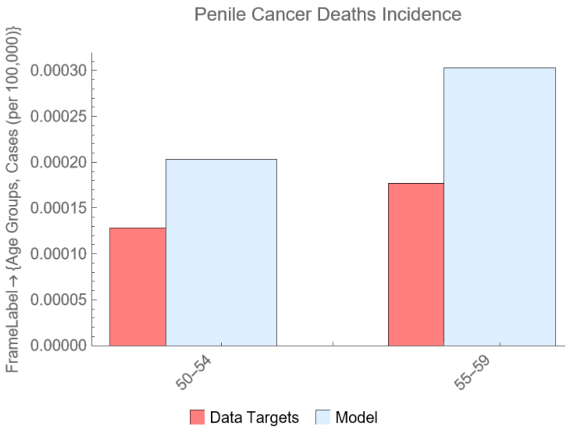

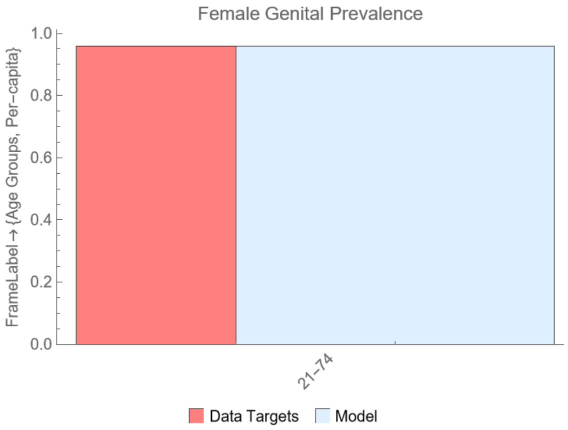

Fit for HPV31 cancer and mortality incidence and female genital prevalence

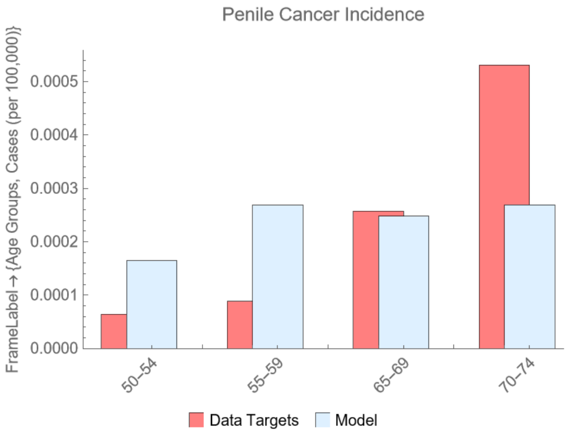

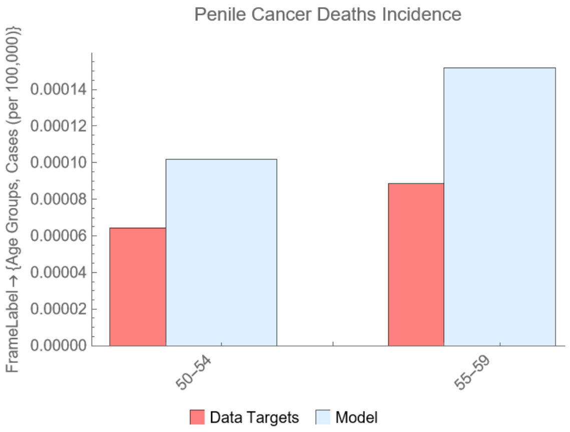

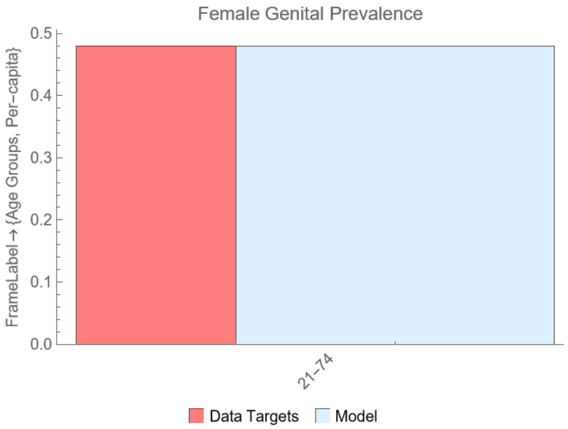

Fit for HPV33 cancer and mortality incidence and female genital prevalence

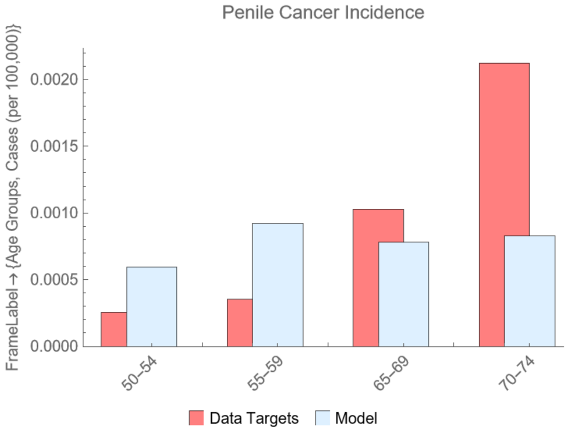

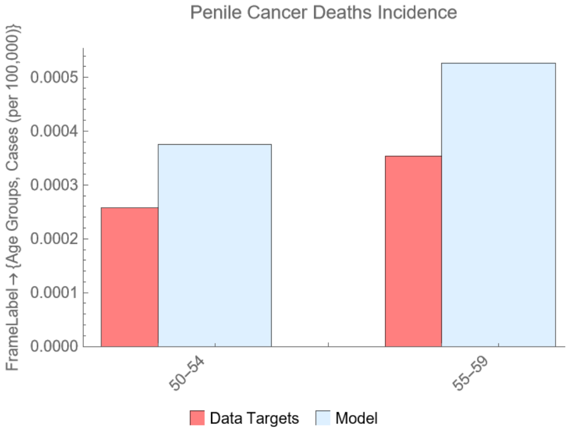

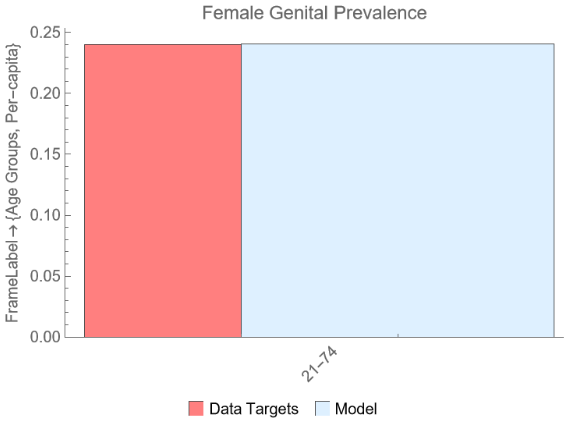

Fit for HPV45 cancer and mortality incidence and female genital prevalence

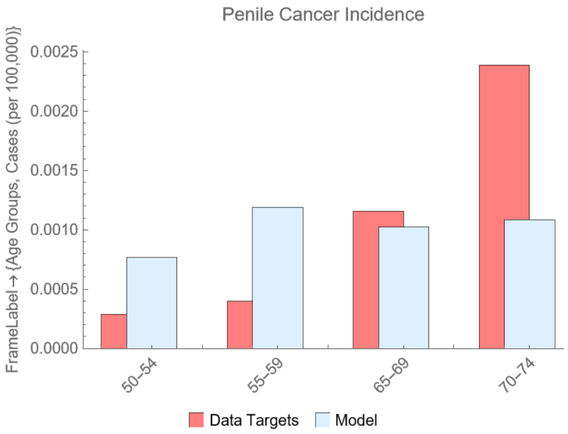

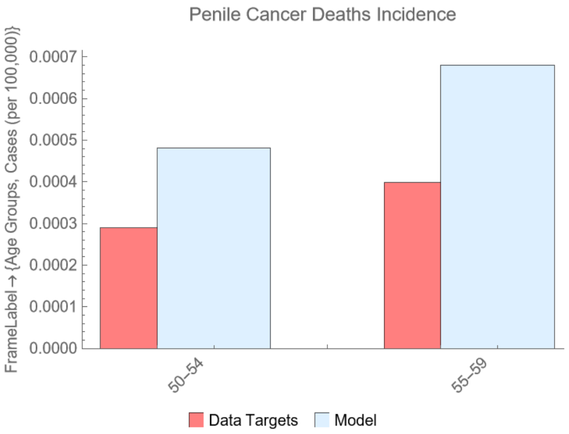

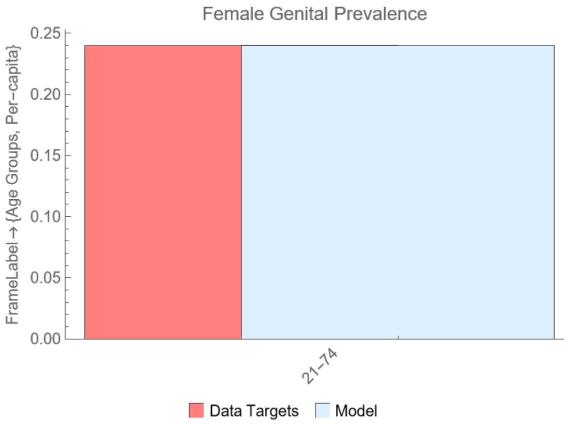

Fit for HPV52 cancer and mortality incidence and female genital prevalence

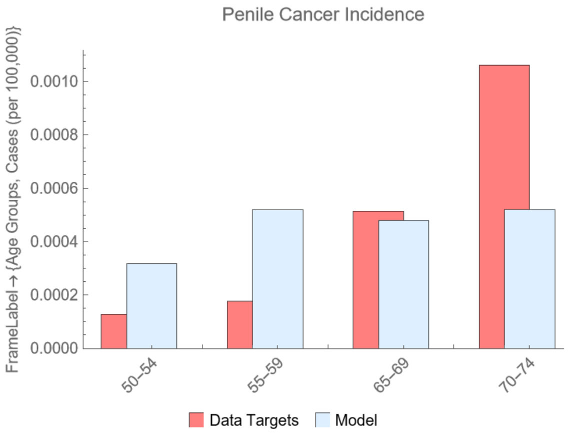

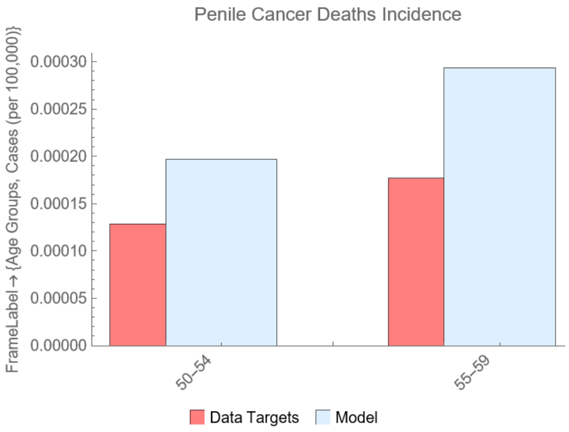

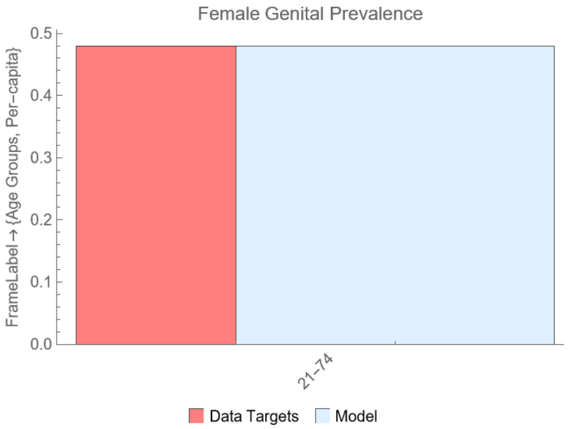

Fit for HPV58 cancer and mortality incidence and female genital prevalence

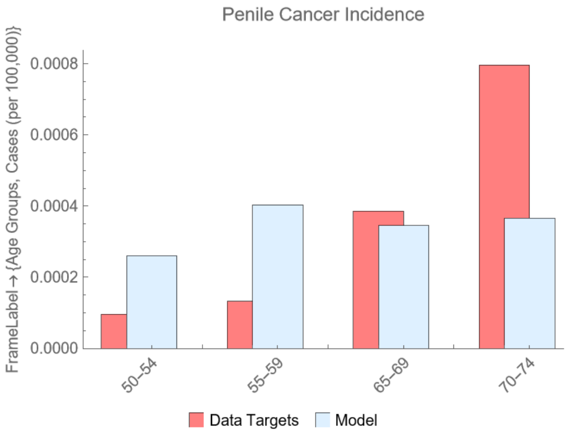

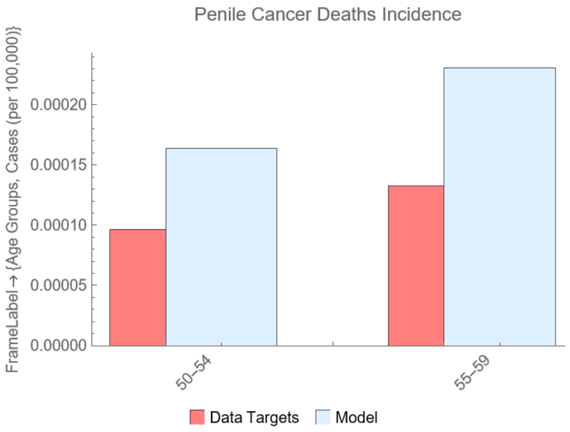

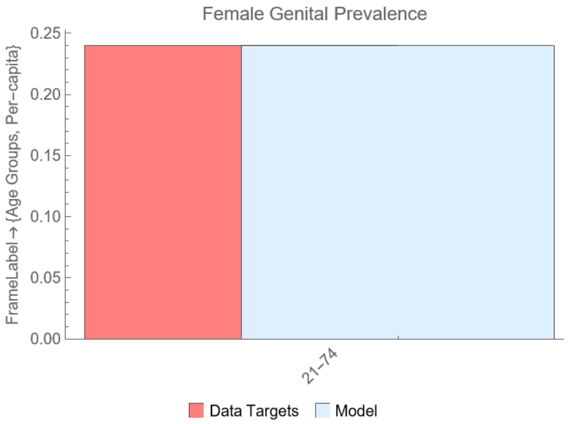

Vaginal

Fit for HPV 16 cancer and mortality incidence and female genital prevalence

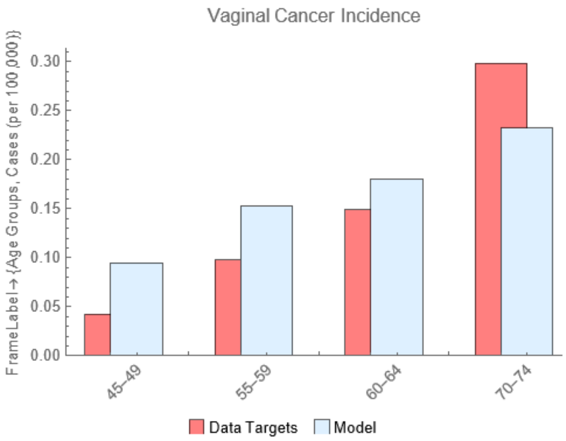

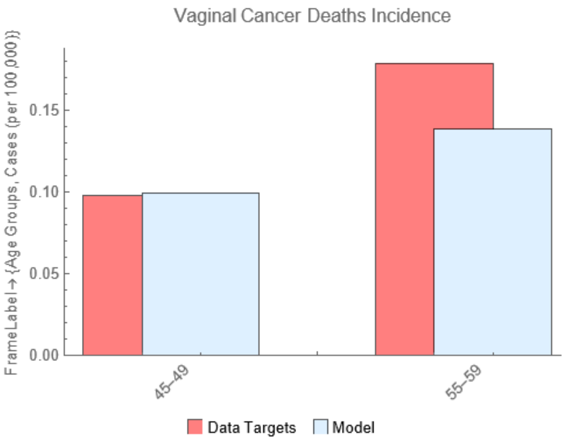

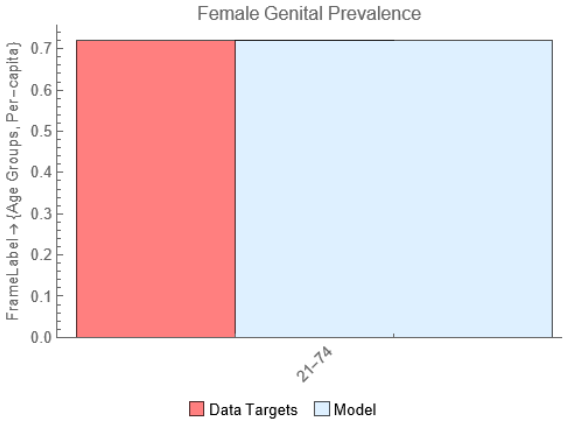

Fit for HPV18 cancer and mortality incidence and female genital prevalence

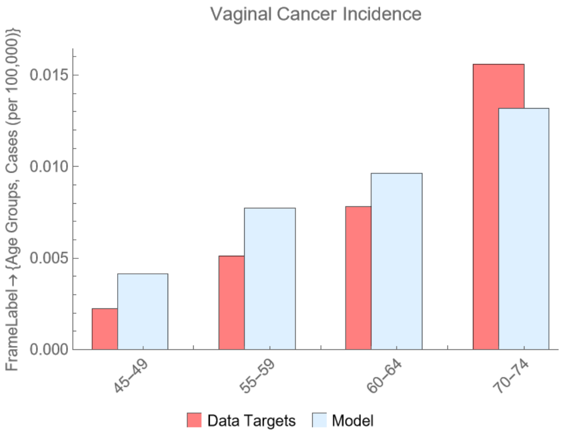

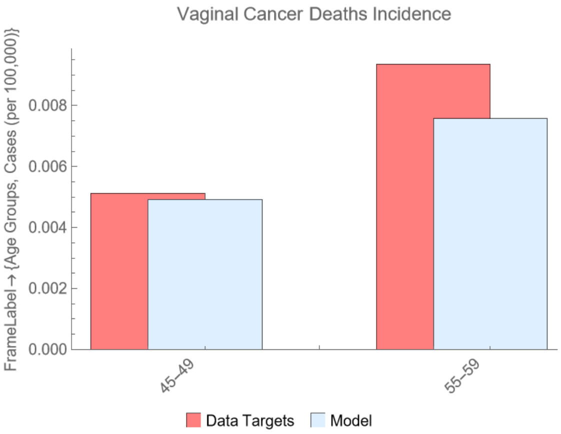

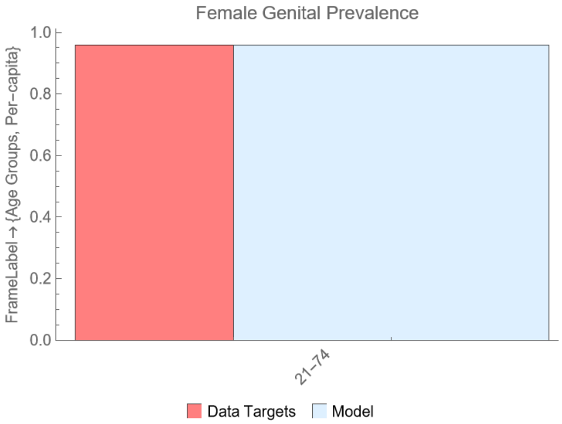

Fit for HPV31 cancer and mortality incidence and female genital prevalence

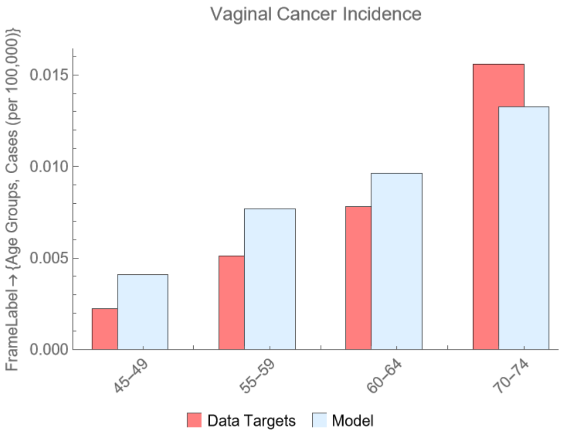

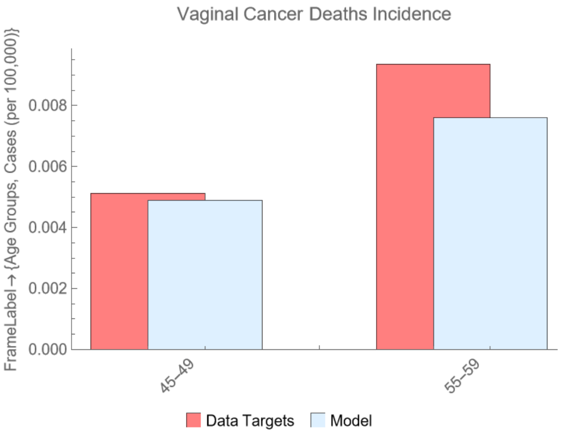

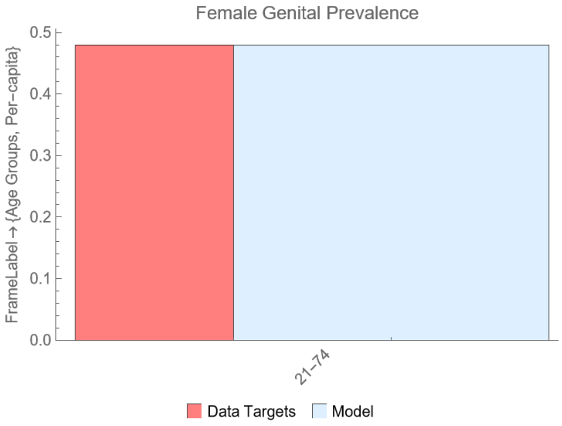

Fit for HPV33 cancer and mortality incidence and female genital prevalence

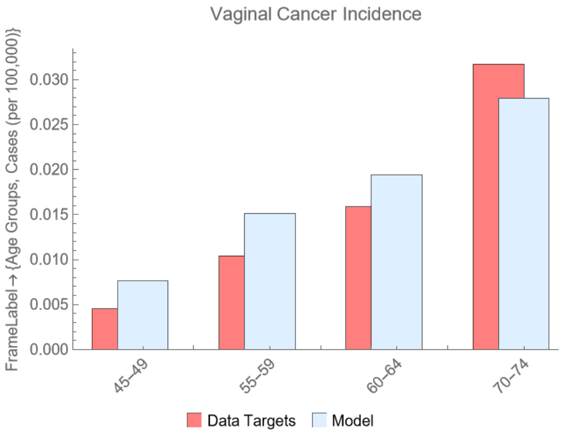

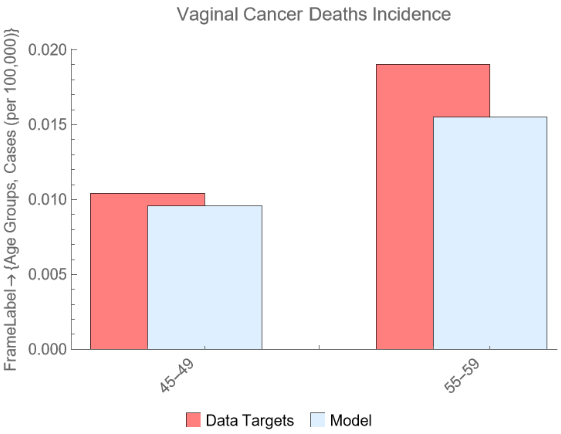

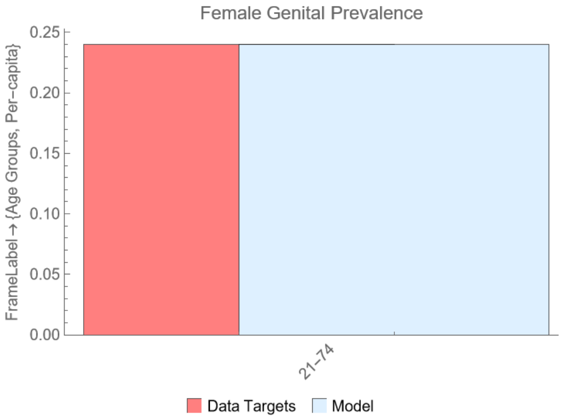

Fit for HPV45 cancer and mortality incidence and female genital prevalence

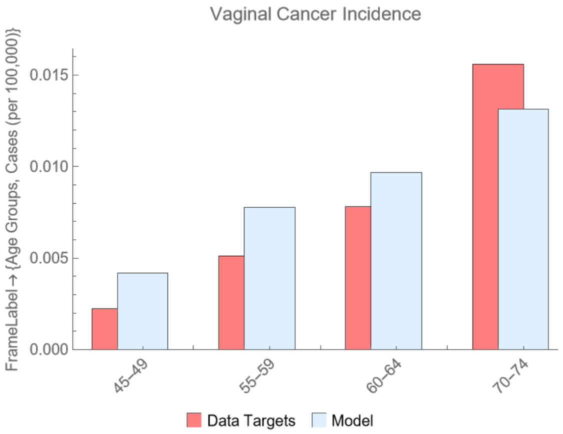

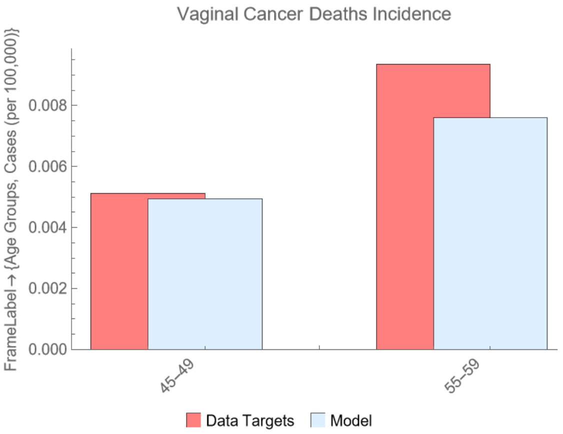

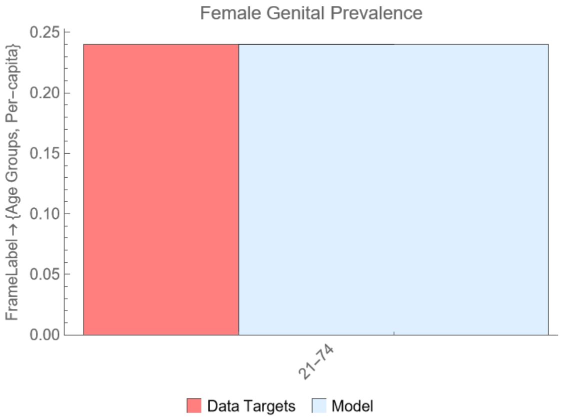

Fit for HPV52 cancer and mortality incidence and female genital prevalence

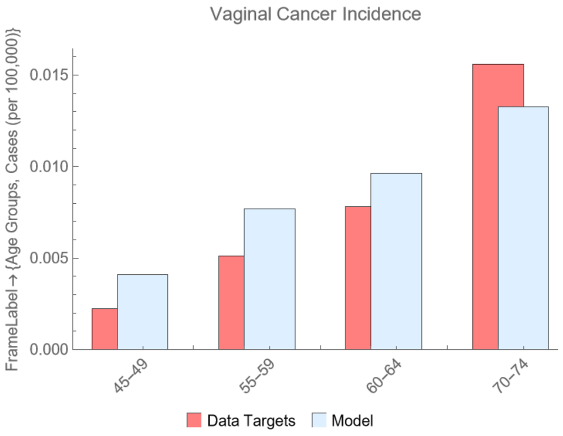

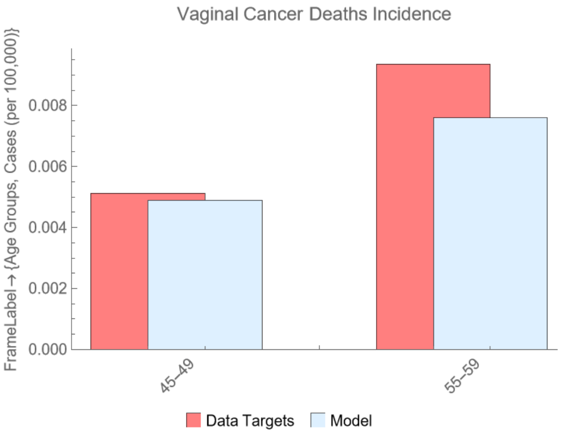

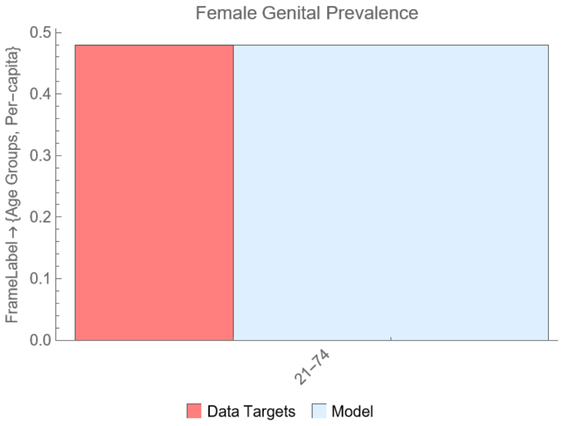

Fit for HPV58 cancer and mortality incidence and female genital prevalence

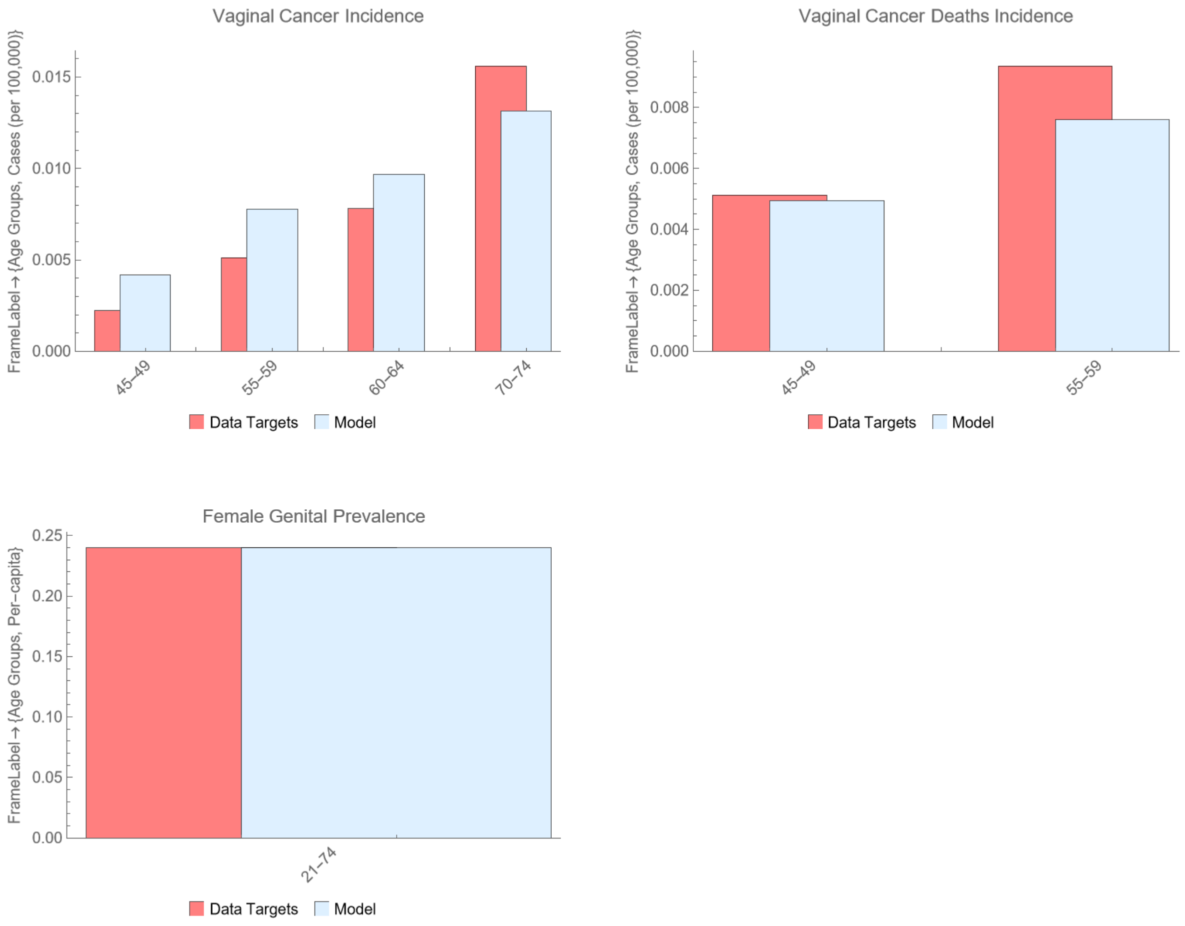

Vulvar

Vulvar model fit results. Note there is no HPV31 attribution for vulvar cancer.

Fit for HPV 16 cancer and mortality incidence and female genital prevalence

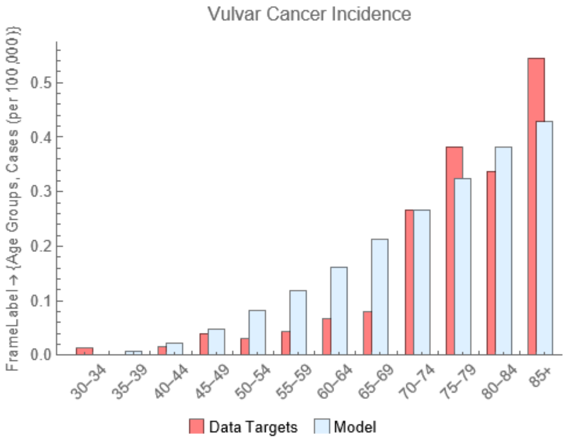

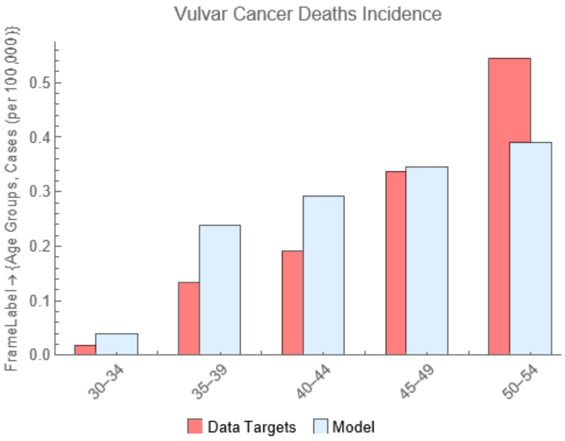

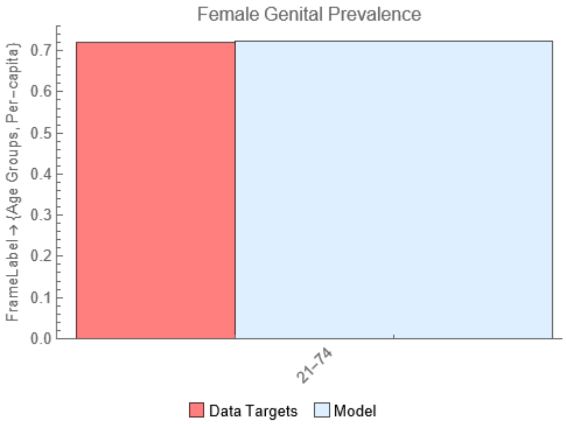

Fit for HPV18 cancer and mortality incidence and female genital prevalence

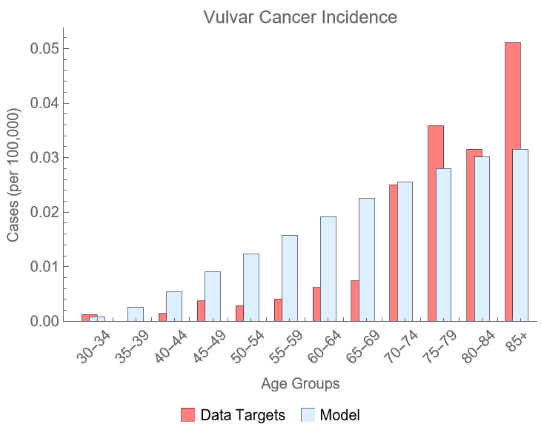

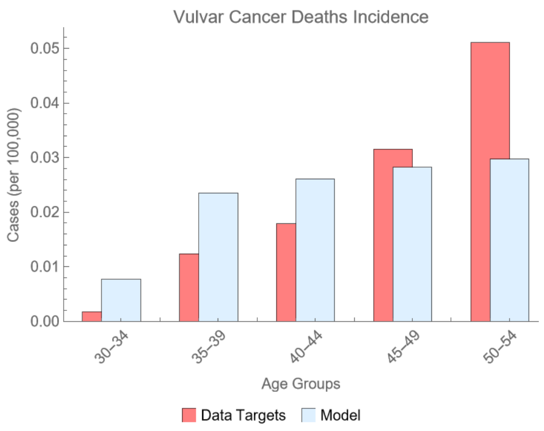

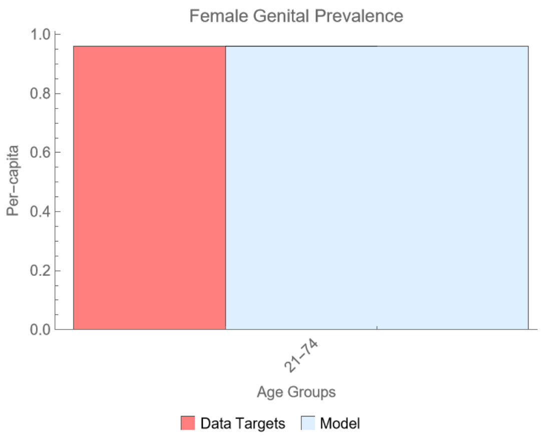

Fit for HPV33 cancer and mortality incidence and female genital prevalence

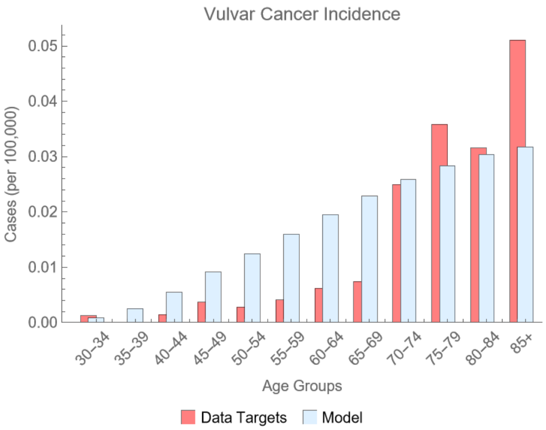

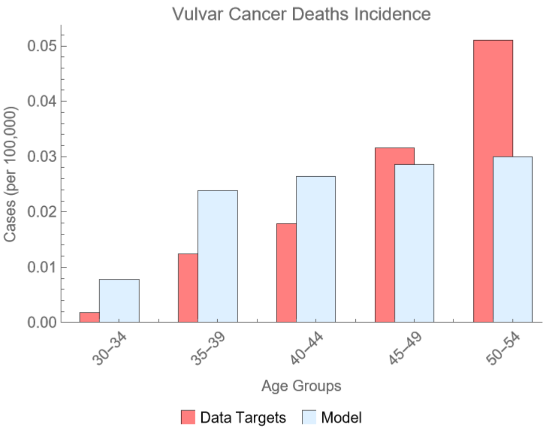

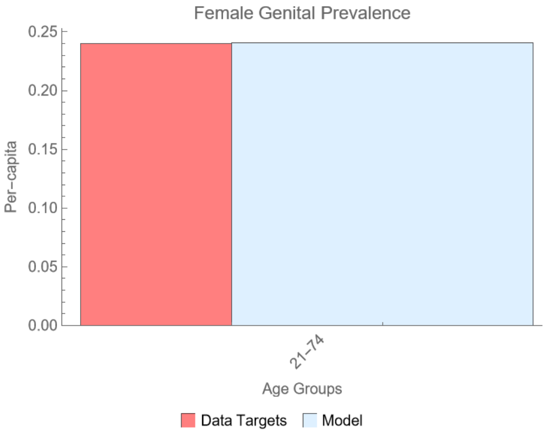

Fit for HPV45 cancer and mortality incidence and female genital prevalence

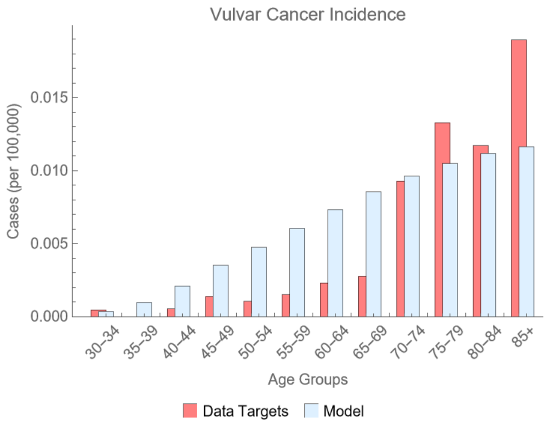

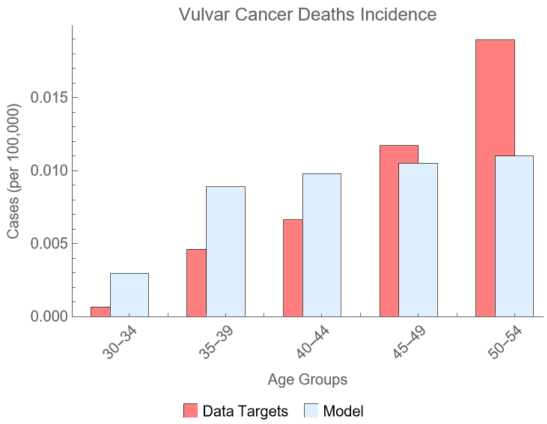

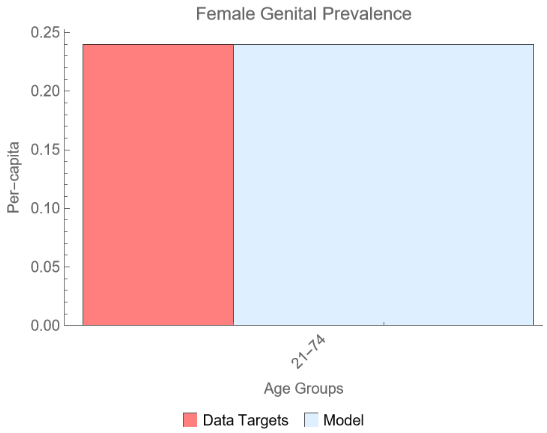

Fit for HPV52 cancer and mortality incidence and female genital prevalence

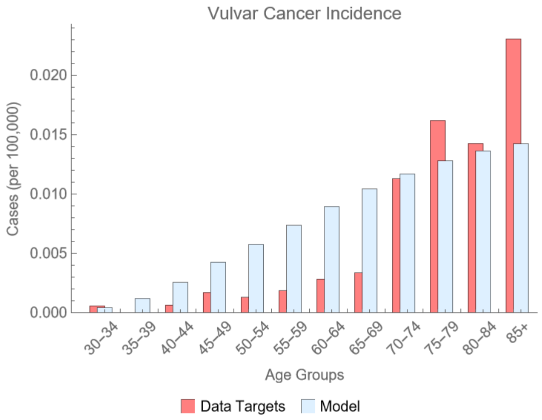

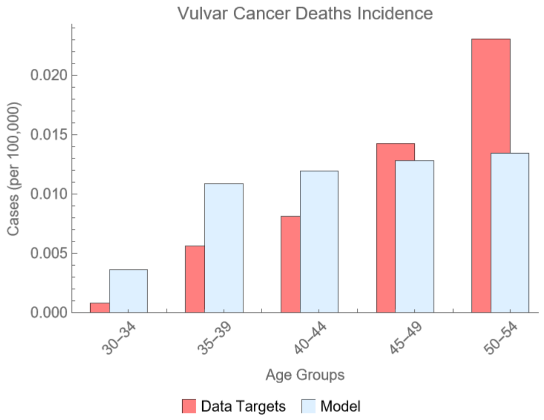

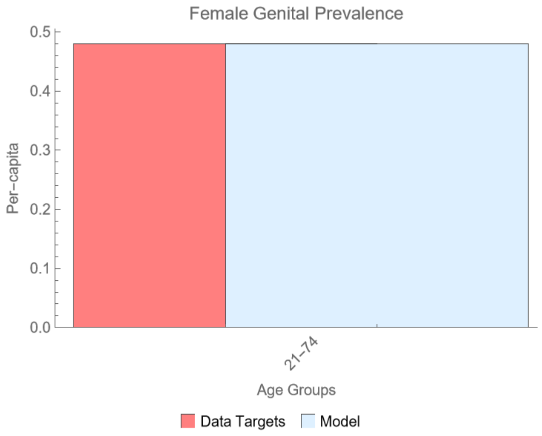

Fit for HPV58 cancer and mortality incidence and female genital prevalence

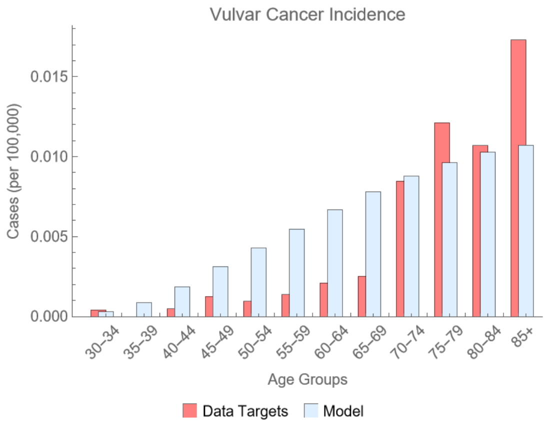

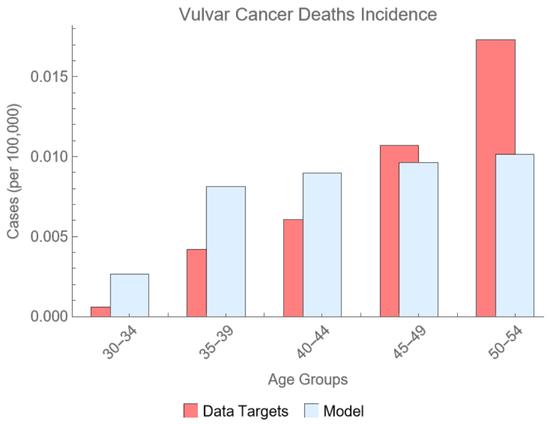

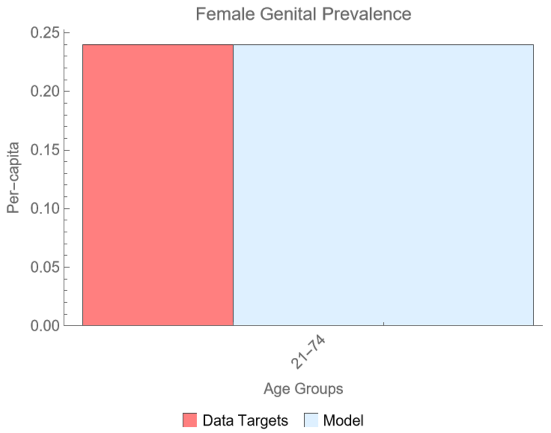

Deterministic sensitivity analysis

S12. Tornado diagram showing the influence of various input parameters on the mean ICER.

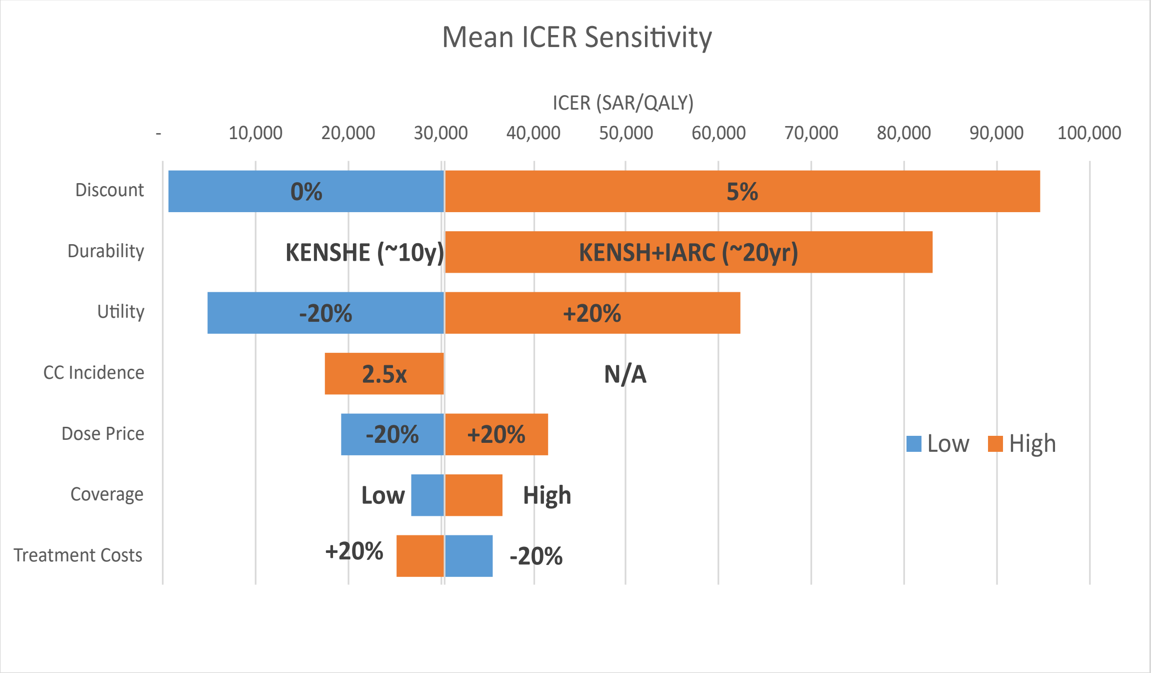

CC, cervical cancer; DSA, deterministic sensitivity analysis; ICER, incremental cost-effectiveness ratio; QALY, quality-adjusted life year

References

[1] World Health Organization (WHO). Life tables by country, Saudi Arabia, https://apps.who.int/gho/data/view.searo.61440?lang=en; 2020 [accessed July 22, 2024,

[2] Raheel H, Mahmood MA, BinSaeed A. Sexual practices of young educated men: implications for further research and health education in Kingdom of Saudi Arabia (KSA). J Public Health (Oxf) 2013;35:(1). 10.1093/pubmed/fds055.

[3] Alhamlan FS, Khayat HH, Ramisetty-Mikler S, Al-Muammar TA, Tulbah AM, Al-Badawi IA, et al. Sociodemographic characteristics and sexual behavior as risk factors for human papillomavirus infection in Saudi Arabia. Int J Infect Dis 2016;4610.1016/j.ijid.2016.04.004.

[4] de Sanjose S, Quint WG, Alemany L, Geraets DT, Klaustermeier JE, Lloveras B, et al. Human papillomavirus genotype attribution in invasive cervical cancer: a retrospective cross-sectional worldwide study. Lancet Oncol 2010;11:(11). 10.1016/S1470-2045(10)70230-8.

[5] Alemany L, Saunier M, Tinoco L, Quiros B, Alvarado-Cabrero I, Alejo M, et al. Large contribution of human papillomavirus in vaginal neoplastic lesions: a worldwide study in 597 samples. Eur J Cancer 2014;50:(16). 10.1016/j.ejca.2014.07.018.

[6] Serrano B, de Sanjose S, Tous S, Quiros B, Munoz N, Bosch X, et al. Human papillomavirus genotype attribution for HPVs 6, 11, 16, 18, 31, 33, 45, 52 and 58 in female anogenital lesions. Eur J Cancer 2015;51:(13). 10.1016/j.ejca.2015.06.001.
[7] Alemany L, Saunier M, Alvarado-Cabrero I, Quiros B, Salmeron J, Shin HR, et al. Human papillomavirus DNA prevalence and type distribution in anal carcinomas worldwide. Int J Cancer 2015;136:(1). 10.1002/ijc.28963.
[8] Alemany L, Cubilla A, Halec G, Kasamatsu E, Quiros B, Masferrer E, et al. Role of Human Papillomavirus in Penile Carcinomas Worldwide. Eur Urol 2016;69:(5). 10.1016/j.eururo.2015.12.007.
[9] Castellsague X, Alemany L, Quer M, Halec G, Quiros B, Tous S, et al. HPV Involvement in Head and Neck Cancers: Comprehensive Assessment of Biomarkers in 3680 Patients. J Natl Cancer Inst 2016;108:(6). 10.1093/jnci/djv403.
[10] Alkhalawi E, Allemani C, Al-Zahrani AS, Coleman MP. Cervical cancer in Saudi Arabia: trends in survival by stage at diagnosis and geographic region. Annals of Cancer Epidemiology 2022;610.21037/ace-22-2.
[11] US Surveillance Epidemiology and End Results (SEER) Program. Public-Use Data (1973–2002), https://seer.cancer.gov/; [accessed November 19, 2024,
[12] National Health Service United Kingdom (NHS). Cancer Survival in England, cancers diagnosed 2016 to 2020, followed up to 2021, https://digital.nhs.uk/data-and-information/publications/statistical/cancer-survival-in-england/cancers-diagnosed-2016-to-2020-followed-up-to-2021/#; 2023 [accessed July 23, 2024,
[13] Alsbeih G, Al-Harbi N, Bin Judia S, Al-Qahtani W, Khoja H, El-Sebaie M, et al. Prevalence of Human Papillomavirus (HPV) Infection and the Association with Survival in Saudi Patients with Head and Neck Squamous Cell Carcinoma. Cancers (Basel) 2019;11:(6). 10.3390/cancers11060820.
[14] International Agency for Research on Cancer (IARC). Saudi Arabia Human Papillomavirus and Related Cancers Fact Sheet 2023, https://hpvcentre.net/statistics/reports/SAU_FS.pdf; 2023 [accessed October 29, 2024,
[15] Bagga R, Singla R, Srinivasan R, Singh T, Verma M. Experience with a novel community outreach approach to cervical cancer screening by colposcopy in the mobile unit. BMJ Innovation 2025;1110.1136/bmjinnov-2024-001277.
[16] Bigras G, de Marval F. The probability for a Pap test to be abnormal is directly proportional to HPV viral load: results from a Swiss study comparing HPV testing and liquid-based cytology to detect cervical cancer precursors in 13,842 women. Br J Cancer 2005;93:(5). 10.1038/sj.bjc.6602728.
[17] Coste J, Cochand-Priollet B, de Cremoux P, Le Gales C, Cartier I, Molinie V, et al. Cross sectional study of conventional cervical smear, monolayer cytology, and human papillomavirus DNA testing for cervical cancer screening. BMJ 2003;326:(7392). 10.1136/bmj.326.7392.733.
[18] Mitchell MF, Schottenfeld D, Tortolero-Luna G, Cantor SB, Richards-Kortum R. Colposcopy for the diagnosis of squamous intraepithelial lesions: a meta-analysis. Obstet Gynecol 1998;91:(4). 10.1016/s0029-7844(98)00006-4.
[19] Kumari P, Kundu J. Prevalence, socio-demographic determinants, and self-reported reasons for hysterectomy and choice of hospitalization in India. BMC Womens Health 2022;22:(1). 10.1186/s12905-022-02072-7.

[20] Alsaqa'aby M, Mamane C, Ugrekhelidze D, Jose J, Sahabote V, Peer P. EE708 Assessment of Direct Costs of Human Papillomavirus Related Diseases in Saudi Arabia. Value in Health 2024;27:(12, S194).
[21] Szende A, Williams A. Measuring self-reported population health: SpringMed Publishing Ltd; 2004.
[22] Insinga RP, Glass AG, Myers ER, Rush BB. Abnormal outcomes following cervical cancer screening: event duration and health utility loss. Med Decis Making 2007;27:(4). 10.1177/0272989X07302128.
[23] Myers ER, Green S, Lipkus I. Patient preferences for health states related to HPV infection: visual analog scales vs time trade-off elicitation (Abstract 390.2). 21st International Papillomavirus Conference; 2004; Mexico City, Mexico.
[24] Gold MR, Franks P, McCoy KI, Fryback DG. Toward consistency in cost-utility analyses: using national measures to create condition-specific values. Med Care 1998;36:(6). 10.1097/00005650-199806000-00002.
[25] Wenzel L, DeAlba I, Habbal R, Kluhsman BC, Fairclough D, Krebs LU, et al. Quality of life in long-term cervical cancer survivors. Gynecol Oncol 2005;97:(2). 10.1016/j.ygyno.2005.01.010.
[26] Lindman JP, Lewis LS, Accortt N, Wiatrak BJ. Use of the Pediatric Quality of Life Inventory to assess the health-related quality of life in children with recurrent respiratory papillomatosis. Ann Otol Rhinol Laryngol 2005;114:(7). 10.1177/000348940511400701.
[27] International Agency for Research on Cancer (IARC). HPV Information Centre, https://hpvcentre.net/parser.php?xml=M2_Cervical%20Cancer_Incidence%20rates&iso=SAU&title=M2.%20Disease%20burden%20estimates%20-%20Cervical%20cancer%20-%20Incidence%20rates; 2024 [accessed July 23, 2024,
